# CircCDYL2 bolsters radiotherapy resistance in nasopharyngeal carcinoma by promoting RAD51 translation initiation for enhanced homologous recombination repair

**DOI:** 10.1186/s13046-024-03049-0

**Published:** 2024-04-23

**Authors:** Hongke Qu, Yumin Wang, Qijia Yan, Chunmei Fan, Xiangyan Zhang, Dan Wang, Can Guo, Pan Chen, Lei Shi, Qianjin Liao, Ming Zhou, Fuyan Wang, Zhaoyang Zeng, Bo Xiang, Wei Xiong

**Affiliations:** 1 NHC Key Laboratory of Carcinogenesis and Hunan Key Laboratory of Cancer Metabolism, Hunan Cancer Hospital and the Affiliated Cancer Hospital of Xiangya School of Medicine, Central South University, Changsha, 410078 China; 2 Key Laboratory of Carcinogenesis and Cancer Invasion of the Chinese Ministry of Education, Cancer Research Institute and School of Basic Medical Sciences, Central South University, Changsha, Hunan 410078 China; 3 Department of Otolaryngology Head and Neck Surgery, Xiangya Hospital, Central South University, Changsha, Hunan 410078 China; 4 National Clinical Research Center for Geriatric Disorders, Xiangya Hospital, Central South University, Changsha, Hunan 410078 China; 5 Department of Pathology, the Second Xiangya Hospital, Central South University, Changsha, Hunan 410011 China

**Keywords:** Nasopharyngeal carcinoma, circCDYL2, RAD51, Homologous recombination repair, Radiotherapy resistance

## Abstract

**Background:**

Radiation therapy stands to be one of the primary approaches in the clinical treatment of malignant tumors. Nasopharyngeal Carcinoma, a malignancy predominantly treated with radiation therapy, provides an invaluable model for investigating the mechanisms underlying radiation therapy resistance in cancer. While some reports have suggested the involvement of circRNAs in modulating resistance to radiation therapy, the underpinning mechanisms remain unclear.

**Methods:**

RT-qPCR and in situ hybridization were used to detect the expression level of circCDYL2 in nasopharyngeal carcinoma tissue samples. The effect of circCDYL2 on radiotherapy resistance in nasopharyngeal carcinoma was demonstrated by in vitro and in vivo functional experiments. The HR-GFP reporter assay determined that circCDYL2 affected homologous recombination repair. RNA pull down, RIP, western blotting, IF, and polysome profiling assays were used to verify that circCDYL2 promoted the translation of RAD51 by binding to EIF3D protein.

**Results:**

We have identified circCDYL2 as highly expressed in nasopharyngeal carcinoma tissues, and it was closely associated with poor prognosis. In vitro and in vivo experiments demonstrate that circCDYL2 plays a pivotal role in promoting radiotherapy resistance in nasopharyngeal carcinoma. Our investigation unveils a specific mechanism by which circCDYL2, acting as a scaffold molecule, recruits eukaryotic translation initiation factor 3 subunit D protein (EIF3D) to the 5′-UTR of RAD51 mRNA, a crucial component of the DNA damage repair pathway to facilitate the initiation of RAD51 translation and enhance homologous recombination repair capability, and ultimately leads to radiotherapy resistance in nasopharyngeal carcinoma.

**Conclusions:**

These findings establish a novel role of the circCDYL2/EIF3D/RAD51 axis in nasopharyngeal carcinoma radiotherapy resistance. Our work not only sheds light on the underlying molecular mechanism but also highlights the potential of circCDYL2 as a therapeutic sensitization target and a promising prognostic molecular marker for nasopharyngeal carcinoma.

**Supplementary Information:**

The online version contains supplementary material available at 10.1186/s13046-024-03049-0.

## Introduction

Nasopharyngeal Carcinoma (NPC) is a malignant tumor originating from the nasopharyngeal epithelium [1]. Its incidence demonstrates distinct geographical variations, with Southeast Asia, South China, and North Africa being high-risk regions [2, 3]. Genetic susceptibility and environmental factors such as EBV viral infection, contribute jointly to the development of nasopharyngeal carcinoma [4]. Radiotherapy stands as the primary clinical treatment approach for nasopharyngeal carcinoma due to its relative sensitivity to ionizing radiation. However, despite its effectiveness in achieving local control, some cancer cells exhibit resistance to radiation therapy. This leads to the persistence of cancer cells, local recurrence, distant metastasis, and ultimately treatment failure and patient mortality [5–7].

Radiation therapy induces DNA double-strand breaks (DSBs), which represent one of the most severe forms of DNA damage and can lead to genomic instability, cell cycle arrest, and apoptosis, among other catastrophic cellular events [8–10]. Cells possess multiple DNA damage repair systems, and successful repair of DNA damage enables cell survival, contributing to radiotherapy resistance. Cellular DNA double-strand break repair predominantly engages two main pathways: homologous recombination (HR) and non-homologous end joining (NHEJ) [11]. HR initiation hinges upon the core protein recombinase RAD51, which is recruited to DNA double-strand break ends by the BRCA1-PALB2-BRCA2 complex to displace RPA. HR utilizes the undamaged homologous sister chromatid as a template for precise repair [12, 13]. Conversely, NHEJ directly ligates the two ends without template involvement, facilitated by proteins such as Ku70-Ku80 and DNA-PKcs [14, 15]. Despite advancements, many aspects of the DNA damage repair pathways remain elusive. Understanding the underlying biological mechanisms of nasopharyngeal carcinoma radiotherapy resistance is paramount for identifying biomarkers and optimizing clinical treatment strategies for nasopharyngeal carcinoma diagnosis, prognosis, and therapy.

Circular RNA (circRNA) represents a novel class of non-coding RNA that has garnered significant attention in recent years. CircRNA is widely implicated in the progression of nasopharyngeal carcinoma, functioning either as an oncogene or a tumor suppressor gene. Our team has previously identified numerous circRNAs involved in the pathogenesis and progression of nasopharyngeal carcinoma, exerting influence on invasion, migration, immune evasion, and radiotherapy resistance [16–22]. Moreover, circRNA plays a crucial role in mediating the tumor’s resistance to radiotherapy, making it a potential molecular target for enhancing radiotherapy sensitivity, as well as serving as clinical diagnostic and prognostic indicators [23, 24]. Till now, the molecular mechanisms underpinning radiotherapy resistance in nasopharyngeal carcinoma remain poorly understood. Notably, the emerging role of circRNAs in radiotherapy resistance warrants extensive exploration.

In this study, we report for the first time that circCDYL2 is highly expressed in nasopharyngeal carcinoma tissues and is closely associated with poor patient prognosis. In vitro and in vivo experiments confirm that circCDYL2 promotes radiotherapy resistance of nasopharyngeal carcinoma. A detailed analysis unravels the mechanism by which circCDYL2 enhances the capacity of nasopharyngeal carcinoma cells to repair DNA damage through homologous recombination following exposure to ionizing radiation. It achieves this by facilitating the translation initiation of RAD51, a key recombinase, ultimately culminating in radiotherapy resistance.

## Materials and methods

### Cell lines

The nasopharyngeal carcinoma cell lines, HNE2 and CNE2, utilized in this study were obtained from the Cancer Research Institute, Central South University. These cells were cultured in a DMEM medium (Life Technologies, NY, USA) supplemented with 10% fetal bovine serum (Gibco, MA, USA) and 1% penicillin-streptomycin (Life Technologies, NY, USA). All cell cultures were maintained at 37 °C in a 5% CO2 humidified incubator.

### Clinical nasopharyngeal carcinoma samples

In this study, two sets of clinical samples were employed. The first set comprised 45 fresh nasopharyngeal carcinoma tissues, while 23 control nasopharyngeal epithelium (NPE) samples were collected from either Xiangya Hospital or the Affiliated Cancer Hospital of Xiangya School of Medicine of Central South University for RT-qPCR detection of circCDYL2 expression. Further clinical information is detailed in Supplemental Table 1. The second set consisted of paraffin-embedded tissue section samples from 203 newly diagnosed nasopharyngeal carcinoma patients, including 102 from Xiangya Hospital and 101 from the Affiliated Cancer Hospital of Xiangya School of Medicineof Central South University. Among these samples, there were 56 also contained adjacent non-cancerous epithelial tissues. All tissue samples were pathologically examined and independently confirmed. Following diagnosis, these patients received standard radiotherapy and were closely monitored accordingly. Comprehensive clinical information is listed in Supplemental Table 2. This research was approved by the Central South University Ethics Committee, and all participants provided informed consent.

### Fluorescence in situ hybridization and in situ hybridization

Fluorescence in situ hybridization (FISH) and in situ hybridization (ISH) analyses were conducted using the in situ hybridization kit (BOSTER, Wuhan, China) according to the manufacturer’s instructions. Dig-labeled probes (Sangon Biotech, Shanghai, China) were used to detect circCDYL2 expression in nasopharyngeal carcinoma cells or clinical tissue samples. FISH employed fluorescence secondary antibody (Invitrogen, CA, USA), while ISH used DAB staining reagents (Meixin Biology, Fujian, China). Cell nuclei were counterstained with DAPI (Invitrogen, CA, USA, for FISH) or hematoxylin (for ISH). FISH slides were observed and photographed using confocal laser scanning microscopy (Perkin Elmer, MA, USA). The ISH slides were independently evaluated by two pathologists and analyzed using a semi-quantitative integral method. The sequences of circCDYL2 probes used in this study are provided in Supplemental Table 3.

### Plasmids, siRNA, and cell transfection

The plasmid for overexpressing circCDYL2 was generated by cloning the second exon of the CDYL2 gene into the circular RNA expression vector pCirc. The EIF3D pcDNA3.1-3xFlag-C overexpression plasmid (NM_003753) was purchased from YouBio Biotech (Guangzhou, China), while the RAD51 overexpression plasmid (NM_002875) was purchased from Weizhen Biotech Co., Ltd. (Shandong, China). ASO-circCDYL2 and siRAD51 were purchased from RIBOBIO (Guangzhou, China). SiRNAs according to EIF3D, BRCA1, and 53BP1 genes were purchased from Qiagen Biotech (Beijing, China). Relevant sequences are provided in Supplemental Table 3. The overexpression vector or empty plasmid was transfected into nasopharyngeal carcinoma cells using Neofect DNA transfection reagent (Neofect Biotech, Beijing, China). SiRNA transfection was carried out using Hiperfect (Qiagen, Hilden, Germany).

### RNA extraction and RT-qPCR quantification

Total RNA was extracted using the Trizol reagent (Solarbio Life Sciences, Shanghai, China) according to the manufacturer’s instructions. The RNA was reverse-transcribed into cDNA using the Vazyme reverse transcription kit (Nanjing, China). Real-time quantitative PCR was performed using SYBR Green (Bimake, Shanghai, China), and GAPDH was used as the internal reference. The primer sequences are listed in Supplemental Table 3.

### Clonogenic assays

Cells that had been transfected with different overexpression vectors or siRNAs were seeded uniformly in 6-well plates. After 24 hours, cells were irradiated with single X-ray doses of 0, 2, 4, 6, and 8 Gy. The cells were then maintained in a cell culture incubator for 8 to 12 days. Then, fixation with 4% paraformaldehyde at room temperature and crystal violet staining was performed. The number of cell colonies was counted for each group, and dose-survival curves were generated for analysis.

### Western blotting

Cell lysis was performed using RIPA buffer (Beyotime Biotechnology, Shanghai, China). Following protein extraction, samples were separated by 10% sodium dodecyl sulfate-polyacrylamide gel electrophoresis (SDS-PAGE) and then transferred to polyvinylidene difluoride (PVDF) membranes (Millipore, Billerica, MA, USA). The membranes were then blocked with 10% skim milk for 1 hour at room temperature and incubated overnight at 4 °C with corresponding primary antibodies. After washing, the membranes were incubated with secondary antibodies for 2 hours at room temperature. Protein bands were detected using ECL reagent (Millipore, Billerica, MA, USA). Quantitative analysis of protein bands was performed using Image J. A list of antibodies used is provided in Supplemental Table 4.

### Cycloheximide and MG132 treatment

The HNE2 and CNE2 cells were treated with cycloheximide (CHX, Beyotime, Shanghai, China) (50 μg/ml) or MG132 (TargetMol, Boston, USA) (20 μM) for 0, 6, 12, or 24 hours, respectively. Then the proteins were extracted for western blotting.

### Immunofluorescence

Cells were washed with PBS, fixed with 4% paraformaldehyde for 20 minutes, and permeabilized with Triton X-100. After that, the cells were blocked with 5% BSA and incubated overnight at 4 °C using the corresponding primary antibodies. Following PBS washing, cells were incubated with appropriate secondary antibodies for 40 minutes at 37 °C. DAPI staining was performed for 10 minutes, then the slides were sealed using a fluorescence quenching attenuator. Images were captured using a confocal laser scanning microscope (Perkin Elmer, MA, USA). Antibodies used are listed in Supplemental Table 4.

### Comet assay

Comet assay was performed using the DNA damage Detection Kit (Jiangsu, China) following the manufacturer’s instructions. In brief, agarose gel was placed on slides, Irradiated cells were digested and resuspended in PBS at a concentration of 1 × 10^6^ /ml, which was mixed with low melting point agarose gel and added to the slides. After solidification, another layer of agarose gel was added. Following incubation in lysis buffer and alkaline buffer, electrophoresis was run at 25 V for 30 minutes in a horizontal electrophoresis apparatus. Propidium lodide (PI) staining was done in the dark for 10 minutes, and DNA migration length was analyzed using a fluorescence microscope.

### HR and NHEJ report assays

The DR-GFP-U2OS and EJ5-GFP-U2OS cells (gifted by Professor Shi Lei, Tianjin Medical University) were used to assess HR and NHEJ repair efficiency, respectively [25]. Cells were seeded in 12-well plates and transfected with the corresponding plasmids or siRNA when they reached 50% confluence. SiBRCA1 and si53BP1 were used as positive controls for HR and NHEJ, respectively. The HA-I-SceI overexpression plasmid was transfected 24 hours later. After an additional 48 hours of incubation, GFP-positive cells were quantified using flow cytometry (Accuri 6, BD Bioscience, New Jersey, USA). At least 20,000 cells were collected for each treatment, and data were analyzed using FlowJo software.

### Polysome profiling assays

Transfected cells were cultured in a medium containing 100 μg/ml of CHX for 5 minutes. After collection, cells were resuspended in cell lysis buffer (5 mM Tris, pH 7.4, 2.5 mM MgCl2, 1.5 mM KCl, 1 × Protease inhibitors, 100 μg/ml CHX, 2 mM DTT, 200 U/mL RNase inhibitor, 0.5% Triton X-100, 0.5% Sodium deoxycholate), centrifuged at 16,000×g, 4 °C for 7 min. Then, 500 ml of supernatant was transferred onto a sucrose gradient (Freshly prepared a 5–50% (w/v) sucrose density gradient in SW41 ultracentrifuge tubes). Centrifugation was carried out at 32,000×g, 4 °C for 2 hours using an ultracentrifuge (Beckman, California, USA). RNA from each fraction was extracted using Trizol (Invitrogen) for RT-qPCR analysis.

### RNA pulldown and liquid chromatography coupled to tandem mass spectrometry

Cells transfected for 48 hours were lysed, and the supernatant was incubated with a biotin-labeled circCDYL2 probe (Sangon Biotech, Shanghai, China; Supplemental Table 3). Streptavidin magnetic beads were added and incubated overnight at 4 °C. After purification, proteins were analyzed using liquid chromatography-tandem mass spectrometry (LC-MS/MS) with an UltiMate 3000 RSLCnano system and LTQ Orbitrap Velos Pro mass spectrometer (Thermo Scientific).

### RNA immunoprecipitation (RIP)

The Magna RIP kit (Millipore, Massachusetts, USA) was used for RIP experiments according to the manufacturer’s instructions. Cells were lysed in lysis buffer and incubated with magnetic beads conjugated to specific target antibodies or negative control IgG antibodies overnight at 4 °C on a rotator. Immunoprecipitated RNA was purified, enriched, and quantified using RT-qPCR. Antibodies used are listed in Supplemental Table 4.

### Immunohistochemistry

Immunohistochemistry was performed using the Elivision™plus (Mouse/Rabbit) Immunohistochemistry Kit (Kit-9902, Maxim, Fujian, China) following the manufacturer’s instructions. Two pathologists independently assessed stained slides. Semi-quantitative integral methods were used for analysis. Antibodies used are listed in Supplemental Table 4.

### Subcutaneous tumor xenografts

Four-week-old female BALB/c nude mice were housed in the SPF center of Hunan Cancer Hospital. Animal experiments (including care, injections, euthanasia, dissection, and sample collection) were conducted following an approved animal experiment protocol. Mice were randomly divided into four groups (*n* = 5), with each mouse being subcutaneously injected with 3 × 10^6^ CNE2 cells transfected with vector, circCDYL2 plasmid, negative control, or ASO-circCDYL2 subcutaneously. Tumor volume and weight were monitored every 2 days. After 18 days, 6 Gy of ionizing radiation was applied to the transplanted tumor site. Mice were observed for an additional 10 days and then euthanized. Tumor tissues were dissected, measured, fixed, paraffin-embedded, and used for subsequent experiments.

### Statistical analysis

Statistical analysis was carried out by using GraphPad Prism 8.0.2. Two-tailed t-tests were used to analyze differences between any two groups, and one-way analysis of variance (ANOVA) was used to analyze differences among multiple groups. All data are presented as mean ± standard deviation (SD), and *p*-values less than 0.05 were considered statistically significant.

## Results

### circCDYL2 is highly expressed in nasopharyngeal carcinoma and associated with poor prognosis

**Fig. 1:**
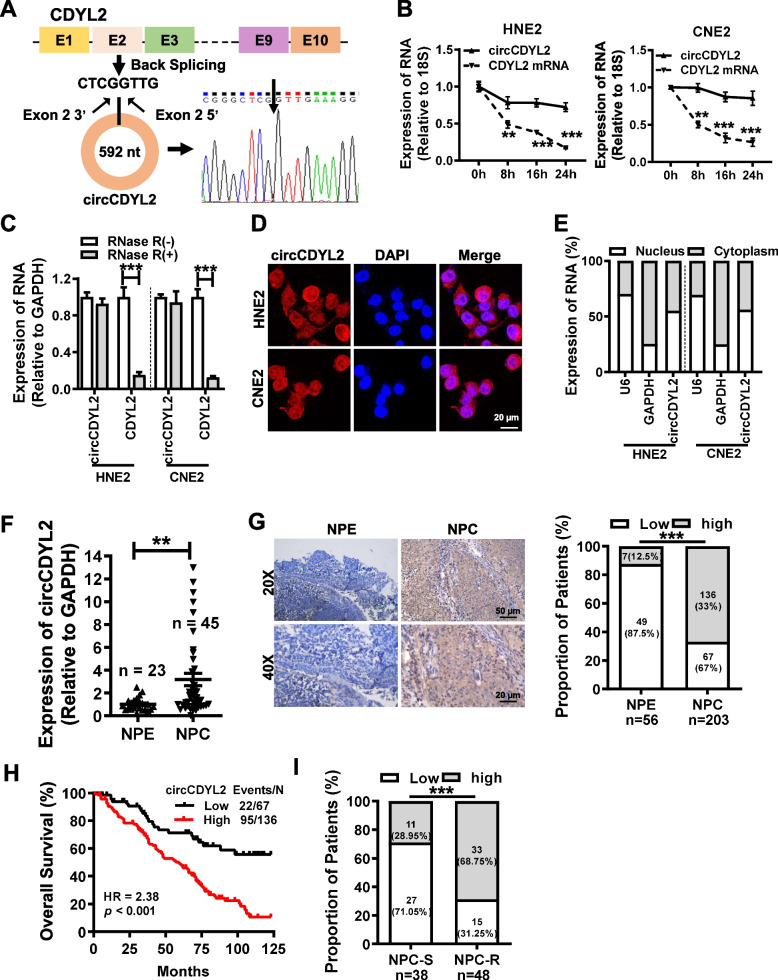
circCDYL2 is highly expressed in nasopharyngeal carcinoma and associated with poor prognosis. **A**. Schematic diagram of the structure of circCDYL2. Sanger sequencing confirmed that circCDYL2 is formed by reverse splicing of exon 2 of CDYL2 pre-mRNA, with a length of 592 nt. **B**. RT-qPCR was used to detect the relative abundance of circCDYL2 and CDYL2 mRNA in HNE2 and CNE2 cells after actinomycin D (1 μg/ml) treatment for 0, 8, 16, or 24 hours. **C**. RT-qPCR was performed to examine the expression of circCDYL2 and CDYL2 mRNA in HNE2 and CNE2 cells after RNase R treatment. **D**. Fluorescence in situ hybridization was used to determine the cellular localization of circCDYL2, showing distribution in both the cytoplasm and the nucleus. Scale bar = 20 μm. **E**. Cellular localization of circCDYL2 was assessed using nuclear-cytoplasmic fractionation followed by RT-qPCR, confirming the presence of circCDYL2 in both cytoplasm and nucleus. **F**. The expression of circCDYL2 was detected by RT-qPCR in fresh nasopharyngeal carcinoma (NPC) biopsy tissues (*n* = 45) and control nasopharyngeal epithelium (NPE) samples (*n* = 23). The expression of circCDYL2 was significantly upregulated in NPC tissues. **G**. In situ hybridization was used to detect circCDYL2 expression levels in 203 archived paraffin-embedded tissues, including 56 adjacent nasopharyngeal epithelium tissues. Results showed that circCDYL2 was highly expressed in 67% (136/203) of NPC tissues compared to 12.5% (7/56) in nasopharyngeal epithelium (NPE) tissues. Representative in situ hybridization results for nasopharyngeal epithelium and NPC tissues are shown; 20×, scale bar = 50 μm; 40×, scale bar = 20 μm. **H**. The overall survival time of NPC patients with high circCDYL2 expression is shorter than that of patients with low circCDYL2 expression, with a five-year survival rate of 48.55% vs 71.32%. HR = 2.38, *p* < 0.001. **I**. The expression of circCDYL2 was significantly higher in the radiotherapy resistance group (NPC_R, *n* = 48), compared with that in the radiotherapy-sensitive group (NPC_S, *n* = 38). All these NPC patients underwent conventional radiotherapy. Patients who did not experience recurrence or metastasis within 5 years after radiotherapy were defined as the radiotherapy-sensitive group (NPC_S, n = 38), while those who experienced recurrence or metastasis within the first 2 years after initial radiotherapy were considered the radiotherapy resistance group (NPC_R, n = 48). Data were represented as mean ± SD. ns, not significant; *, *p* < 0.05; **, *p* < 0.01; ***, *p* < 0.001

In an effort to identify pivotal circRNAs that affect the onset and progression of nasopharyngeal carcinoma, we previously conducted high-throughput RNA sequencing on nasopharyngeal carcinoma cells (NCBI database under submission number: PRJNA391554), unveiling several highly expressed circRNAs within these cells [21]. Among these circRNAs, circCDYL2 has been rarely studied in tumors, and there is no report in nasopharyngeal carcinoma. Sanger sequencing confirmed that circCDYL2 is formed by reverse splicing of exon 2 of CDYL2 (chromodomain Y like 2) pre-mRNA, with a total length of 592 nt (Fig. 1A). Treatment with actinomycin D (Fig. 1B) and RNase R (Fig. 1C) confirmed that circCDYL2 is far more stable than linear CDYL2 mRNA. Fluorescence in situ hybridization with a specific probe targeting the circCDYL2 reverse splicing site (Fig. 1D) and RT-qPCR with fractionated nuclear and cytoplasmic RNA (Fig. 1E) revealed the presence of circCDYL2 in both the nucleus and cytoplasm. Furthermore, a total of 45 fresh nasopharyngeal carcinoma biopsy tissue samples and 23 non-cancer control nasopharyngeal epithelial tissue samples were collected to measure the expression of circCDYL2 (Supplemental Table 1). RT-qPCR analysis revealed significantly elevated expression of circCDYL2 in nasopharyngeal carcinoma tissues (Fig. 1F). Additionally, we obtained 203 archived nasopharyngeal carcinoma paraffin-embedded tissue samples (Supplemental Table 2), of which 56 samples contained adjacent non-cancerous nasopharyngeal epithelium in the same section. In situ hybridization (ISH) experiments also indicated that circCDYL2 was highly expressed in 136 (67%) of nasopharyngeal carcinoma tissues, whereas only 7 (12.5%) of the adjacent non-cancerous epithelium samples exhibited high levels of circCDYL2 expression (Fig. 1G). Crucially, patients with high circCDYL2 expression had significantly lower overall survival rate compared to those with low circCDYL2 expression (5-year survival rate of 48.55% vs 71.32%, HR = 2.38, *p* < 0.001, Fig. 1H). According to the clinical data for these patients, we considered patients who did not experience recurrence or metastasis within 5 years post-radiotherapy as the radiotherapy-sensitive group and patients who experienced recurrence or metastasis within the first 2 years after initial radiotherapy as the radiotherapy resistance group. Notably, the circCDYL2 expression was significantly higher in the radiotherapy resistance group than in the radiotherapy-sensitive group (Fig. 1I). These compelling findings strongly imply a close association between circCDYL2 and nasopharyngeal carcinoma radiotherapy resistance, potentially contributing to poor patient prognoses.

### circCDYL2 enhances the radiotherapy resistance of nasopharyngeal carcinoma by promoting DNA damage repair

**Fig. 2:**
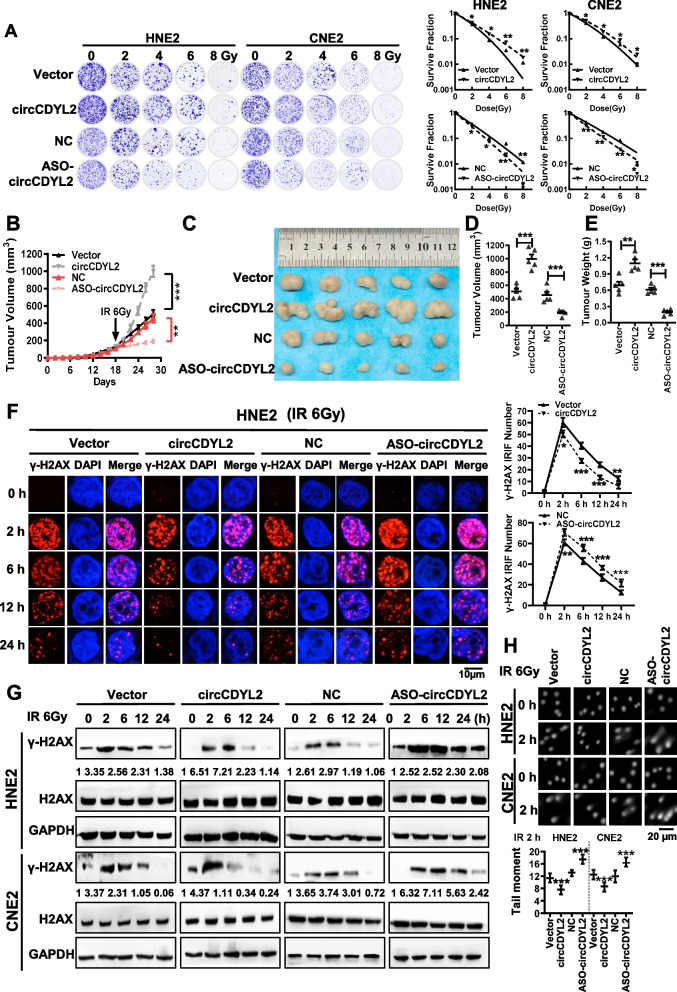
circCDYL2 promotes radiotherapy resistance in vitro and in vivo. **A**. Colony formation assay was performed to measure the survival rates after ionizing radiation in NPC cells. Cells were irradiated with X-rays at doses of 0, 2, 4, 6, or 8 Gy, respectively, after overexpression or knockdown of circCDYL2. The cell survival curve was obtained by using the irradiation dose as the abscissa (arithmetic coordinates), and the survival fraction as the ordinate (logarithmic coordinates). The results were analyzed and plotted using a linear-quadratic model. **B**. In vivo experiments were performed by subcutaneously injecting 3 × 10^6^ CNE2 cells after overexpression or knockdown for circCDYL2 (*n* = 5). Tumor sites were irradiated with 6 Gy on day 18. Tumor volumes were measured every 2 days until day 28. **C**. Representative images of subcutaneous tumors. **D** & **E**. Tumor volumes (**D**) and weights (**E**) were measured in each group. **F**. Immunofluorescence was performed to measure the level of γ-H2AX in HNE2 cells after overexpression or knockdown of circCDYL2 with 6 Gy irradiation for 2, 6, 12, or 24 hours. The results showed that overexpression of circCDYL2 reduced the accumulation of radiation-induced foci (IRIF) of γ-H2AX in HNE2 cells post-irradiation, while knockdown of circCDYL2 had the opposite effect. Scale bar = 10 μm. The right graph represents the number of radiation-induced foci (IRIF) in 30 cells. **G**. Western blotting revealed that circCDYL2 promoted the disappearance of γ-H2AX foci in HNE2 cells with circCDYL2 overexpression or knockdown after irradiation. **H**. Comet assays showed that circCDYL2 promoted DNA damage repair in NPC cells with circCDYL2 overexpression or knockdown after radiation. Representative comet images were shown above, and the scale bar = 20 μm. The lower panel represented statistical analysis. Data were represented as mean ± SD. ns, not significant; *, *p* < 0.05; **, *p* < 0.01; ***, *p* < 0.001

To investigate whether circCDYL2 contributes to radiotherapy resistance in nasopharyngeal carcinoma cells, we exposed CNE2 cells to multiple low-dose radiation treatments, inducing radiotherapy resistance and generating CNE2-IR (radiotherapy resistance) cells. Clonogenic assays confirmed that, following a 2 Gy X-ray irradiation, the number of CNE2-IR cell colonies was significantly greater than that of its parent cell line CNE2 (Supplemental Fig. 1A). RT-qPCR analysis verified that circCDYL2 was significantly upregulated in CNE2-IR cells (Supplemental Fig. 1B). Subsequently, we constructed an overexpression vector for circCDYL2 and designed antisense oligonucleotides (ASOs) targeting the reverse splicing site of circCDYL2. These constructs were transfected into nasopharyngeal carcinoma cells, resulting in either overexpression or knockdown of circCDYL2 (Supplemental Fig. 1C). The cells were then exposed to irradiation with 0, 2, 4, 6, and 8 Gy X-rays. Clonogenic assays revealed that overexpressing circCDYL2 significantly enhanced radiotherapy resistance in nasopharyngeal carcinoma cells, whereas circCDYL2 knockdown increased their sensitivity to ionizing radiation (Fig. 2A).

In addition, we established a subcutaneous tumorigenic model in nude mice by injecting CNE2 cells that either overexpressed or had circCDYL2 knocked down subcutaneously (with cells transfected with blank vector or scramble sequence as controls). After 18 days, subcutaneous tumors had developed, and these tumor-bearing mice received a single 6 Gy radiotherapy treatment. Ten days following radiotherapy, the mice were euthanized, and their tumor volumes were measured. The results demonstrated that, compared to the control group, tumor volumes were notably larger in the circCDYL2 overexpression group and significantly smaller in the circCDYL2 knockdown group (Fig. 2B-E). These in vivo experiments further corroborated that circCDYL2 promotes radiotherapy resistance in nasopharyngeal carcinoma cells.

Ionizing radiation can induce DNA double-strand breaks (DSBs) in cells, which represent a severe form of DNA damage. Unrepaired DSBs can trigger apoptosis, forming the fundamental basis of radiotherapy’s effectiveness in treating malignant tumors. The ability of tumor cells to repair DNA damage is intricately linked to their sensitivity to radiotherapy [26]. Near the sites of DSBs on DNA, H2AX is rapidly phosphorylated to generate γ-H2AX, a widely recognized marker for DSBs [27]. Immunofluorescence assays demonstrated that following exposure to 6 Gy X-rays, cells that overexpressed circCDYL2 exhibited fewer γ-H2AX foci, which also resolved more swiftly, while cells with circCDYL2 knockdown accumulated more γ-H2AX foci. These findings strongly suggest that circCDYL2 can enhance the capacity of DSB repair in nasopharyngeal carcinoma cells (Fig. 2F and Supplemental Fig. 1D). Similar outcomes were obtained through western blotting analysis of γ-H2AX expression levels in cells with circCDYL2 overexpression or knockdown after radiotherapy (Fig. 2G). Furthermore, comet assays also supported that overexpressing circCDYL2 promoted DNA damage repair in nasopharyngeal carcinoma cells following irradiation while knocking down circCDYL2 resulted in more pronounced DNA comet tails (Fig. 2H). Collectively, these results indicate that circCDYL2 boosts DNA damage repair in nasopharyngeal carcinoma cells, thereby promoting radiotherapy resistance.

### circCDYL2 enhances radiotherapy resistance in nasopharyngeal carcinoma by promoting DNA homologous recombination repair

**Fig. 3:**
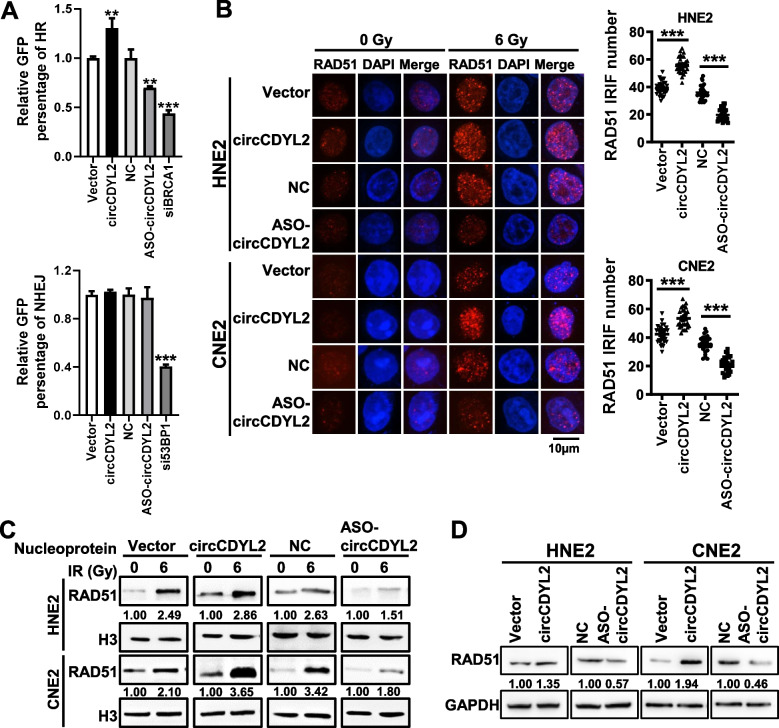
circCDYL2 enhances radiotherapy resistance in nasopharyngeal carcinoma by promoting DNA homologous recombination repair. **A**. Flow cytometry was performed to measure the GFP expression in DR-GFP-U2OS and EJ5-GFP-U2OS cells. Cells were transfected with the overexpression plasmid or ASO of circCDYL2. SiRNAs of BRCA1 or 53BP1 were selected as positive controls. Each group was co-transfected with the HA-Isce1 overexpression plasmid. **B**. Immunofluorescence showed that circCDYL2 enhanced the number of RAD51 irradiation-induced foci (IRIF) in HNE2 and CNE2 cell nuclei after irradiation. Scale bar = 10 μm. On the right, the quantification of IRIF per 30 cells was presented. **C**. The expression of RAD51 in the nucleus after irradiation was detected by western blotting. The results showed that circCDYL2 promoted the accumulation of RAD51 in the nucleus following irradiation. **D**. Western blotting showed that circCDYL2 promoted the expression of RAD51 protein in NPC cells after overexpression or knockdown of circCDYL2. Data were represented as mean ± SD. ns, not significant; *, *p* < 0.05; **, *p* < 0.01; ***, *p* < 0.001

Homologous recombination (HR) and non-homologous end joining (NHEJ) are two major cellular pathways responsible for repairing DNA DSBs [14, 28, 29]. To investigate the mechanism by which circCDYL2 enhances DNA damage repair, thereby contributing to radiotherapy resistance in nasopharyngeal carcinoma, we initially employed two reporter gene systems, DR-GFP and EJ5-GFP (Supplemental Fig. 2A) [30, 31], to assess the capacity for repairing DSBs through HR or NHEJ before and after overexpressing or knocking down circCDYL2. In these experiments, we utilized cells with the knockdown of essential HR protein BRCA1 (breast cancer susceptibility gene 1) and NHEJ’s key protein 53BP1 (tumor protein p53 binding protein 1) as positive controls (Supplemental Fig. 2B). The plasmid overexpressing the restriction endonuclease HA-Isce1 to introduce specific DNA DSBs within the reporter genes were co-transfected into cells. The measurement of GFP expression, as detected by flow cytometry, served as an indicator of the cells’ HR or NHEJ repair capabilities (Supplemental Fig. 2C). The results demonstrated that overexpression of circCDYL2 significantly enhanced HR repair efficiency, whereas knocking down circCDYL2 inhibited HR repair. Importantly, circCDYL2 does not exert any discernible influence on NHEJ repair efficiency (Fig. 3A).

Subsequently, we utilized immunofluorescence to investigate the impact of circCDYL2 on the localization of crucial proteins within the HR pathway at the sites of damage following irradiation. The results revealed that cells overexpressing circCDYL2 displayed a substantial increase in RAD51 foci, the recombinase activated in response to DNA damage. In contrast, cells with circCDYL2 knockdown exhibited a marked reduction in these foci (Fig. 3B). Furthermore, western blotting validated that overexpression of circCDYL2 boosted the localization of RAD51 within the cell nucleus after radiation exposure, while circCDYL2 knockdown resulted in decreased levels of RAD51 protein within the cell nucleus (Fig. 3C). However, circCDYL2 did not have any discernible effect on the aggregation of HR pathway proteins BRCA1 (Supplemental Fig. 3A) and RPA1 (Replication Protein A1) (Supplemental Fig. 3B). Moreover, western blotting unveiled that circCDYL2 promoted the expression levels of RAD51 protein in nasopharyngeal carcinoma cells (Fig. 3D), while it did not influence the expression of BRCA1, RPA1, or Ku70, a key protein within the NHEJ pathway (Supplemental Fig. 3C).

### circCDYL2 enhances RAD51 translation to promote homologous recombination repair in nasopharyngeal carcinoma

**Fig. 4:**
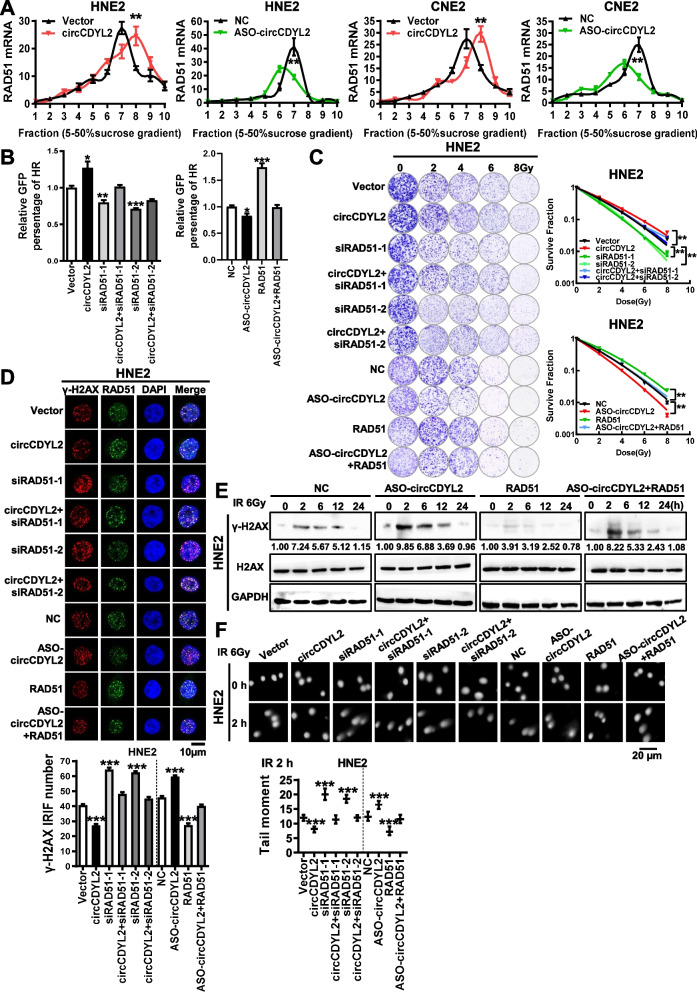
circCDYL2 enhances RAD51 translation to promote DNA homologous recombination repair in nasopharyngeal carcinoma. **A**. Polysome profiling demonstrated that circCDYL2 enhances RAD51 mRNA expression on polysomes in NPC cells after overexpression or knockdown of circCDYL2. **B**. Flow cytometry showed that RAD51 could partially reverse the effect of circCDYL2 on homologous recombination repair in DR-GFP U2OS cells after simultaneous overexpression of circCDYL2 combined with knockdown of RAD51 or knockdown of circCDYL2 with overexpression of RAD51, respectively. Each group was co-transfected with the HA-Isce1 overexpression plasmid. **C**. Clonogenic assays showed that RAD51 partially reversed the effect of circCDYL2 on HNE2 cells survival post-irradiation. Cells were exposed to 0, 2, 4, 6, and 8 Gy X-ray irradiation, respectively after simultaneous overexpression of circCDYL2 combined with knockdown of RAD51 or knockdown of circCDYL2 with overexpression of RAD51. **D**. Immunofluorescence showed that RAD51 could partially reverse the regulation of γ-H2AX radiation-induced foci (IRIF) by circCDYL2 in HNE2 cells These cells were irradiated with 6 Gy X-rays for 6 hours after simultaneous overexpression of circCDYL2 combined with knockdown of RAD51 or knockdown of circCDYL2 with overexpression of RAD51. Scale bar = 10 μm. **E**. Western blotting demonstrated that RAD51 could partially reverse the regulation of γ-H2AX expression levels by circCDYL2 in HNE2 cells. Knockdown of circCDYL2, overexpression of RAD51, and simultaneous knockdown of circCDYL2 with overexpression of RAD51 in HNE2 cells, respectively. These cells were irradiated with 6 Gy X-rays for 0, 2, 6, 12, or 24 hours, respectively. **F**. Comet assays showed that RAD51 mediated the effect of circCDYL2 on DNA damage repair in HNE2 cells after simultaneous overexpression of circCDYL2 combined with knockdown of RAD51 or knockdown of circCDYL2 with overexpression of RAD51, respectively. These cells were irradiated with 6 Gy X-rays, and c Scale bar = 20 μm. Data were represented as mean ± SD. ns, not significant; *, *p* < 0.05; **, *p* < 0.01; ***, *p* < 0.001

To further dissect the mechanism by which circCDYL2 upregulates RAD51, we conducted RT-qPCR experiments and discovered that circCDYL2 had no discernible effect on RAD51 mRNA expression (Supplemental Fig. 4A). Furthermore, treatment with actinomycin D revealed that circCDYL2 did not influence the stability of RAD51 mRNA (Supplemental Fig. 4B). Additionally, we employed cycloheximide (CHX) and MG132 treatments in nasopharyngeal carcinoma cells, and the results indicated that circCDYL2 did not impact the half-life or ubiquitin-mediated degradation of RAD51 (Supplemental Fig. 4C & D). RNA pull-down experiments provided evidence that circCDYL2 did not directly interact with the RAD51 protein (Supplemental Fig. 4E). These findings led us to hypothesize that circCDYL2 likely promotes the translation of RAD51. Polysome profiling assays confirmed that the overexpression of circCDYL2 significantly enhanced the enrichment of RAD51 mRNA on polyribosomes, whereas the knockdown of circCDYL2 produced the opposite effect (Fig. 4A, Supplemental Fig. 5A). These results collectively indicate that circCDYL2 upregulates RAD51 expression by promoting its translation.

To confirm that RAD51 is the mediator of radiotherapy resistance induced by circCDYL2, we simultaneously overexpressed circCDYL2 and knocked down RAD51 in nasopharyngeal carcinoma cells. We employed the DR-GFP reporter system to evaluate the cells’ HR repair capacities. The results demonstrated that the knockdown of RAD51 partially reversed the enhancement of HR repair efficiency induced by circCDYL2 overexpression. Conversely, the overexpression of RAD51 in circCDYL2-knockdown nasopharyngeal carcinoma cells could counteract the reduction in HR repair efficiency caused by circCDYL2 knockdown (Fig. 4B, Supplemental Fig. 5B). Clonogenic assays also unveiled that the knockdown of RAD51 partially reversed the increased clonogenic capacity of cells observed after circCDYL2 overexpression, whereas the expression of RAD51 enhanced the survival of nasopharyngeal carcinoma cells following circCDYL2 knockdown (Fig. 4C, Supplemental Fig. 6A). These findings collectively indicate that circCDYL2 enhances the HR repair capabilities in nasopharyngeal carcinoma cells through upregulation of RAD51, thereby promoting cellular survival after radiotherapy.

In addition, immunofluorescence experiments revealed that the knockdown of RAD51 partially delayed the disappearance of γ-H2AX foci induced by circCDYL2 overexpression, while overexpression of RAD51 partially reduced the prolonged phosphorylation of γ-H2AX resulting from circCDYL2 knockdown (Fig. 4D, Supplemental Fig. 6B). Western blotting analysis to assess γ-H2AX expression yielded results consistent with those from the immunofluorescence experiments (Fig. 4E, Supplemental Fig. 6C). Comet assays further confirmed that the knockdown of RAD51 could impede the DNA damage repair induced by circCDYL2 overexpression, whereas overexpression of RAD51 in circCDYL2-knockdown nasopharyngeal carcinoma cells enhanced DNA damage repair (Fig. 4F, Supplemental Fig. 6D). In summary, these findings collectively suggest that circCDYL2 enhances RAD51 translation, thereby promoting homologous recombination repair in nasopharyngeal carcinoma cells following radiotherapy, ultimately leading to radiotherapy resistance.

**Fig. 5:**
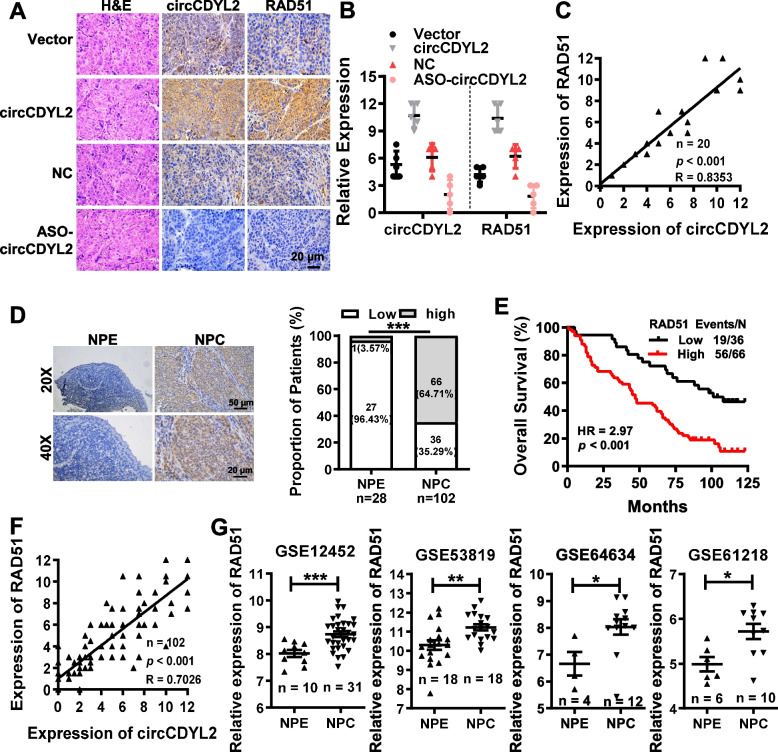
RAD51 is highly expressed in NPC tissues and positively correlated with the expression of circCDYL2. **A**. Representative images of H&E staining, in situ hybridization of circCDYL2, and immunohistochemistry of RAD51 protein using the subcutaneous xenograft tumor tissues. 40×, scale bar = 20 μm. **B**. Statistical results of the relative expression levels of circCDYL2 and RAD51 in the subcutaneous xenograft tumor tissues according to Fig. 5A. **C**. Correlation between circCDYL2 and RAD51 expression was analyzed in the subcutaneous xenograft tumor tissues according to Fig. 5A. R = 0.8353. **D**. Representative immunohistochemistry results of RAD51 protein in 102 paraffin-embedded NPC tissues, compared with 28 adjacent nasopharyngeal epitheliums. 20×, scale bar = 50 μm, and 40×, scale bar = 20 μm. **E**. Overall survival analysis of NPC patients with low and high RAD51 expression using a Kaplan-Meier curve. **F**. Correlation between circCDYL2 and RAD51 expression was analyzed in 102 nasopharyngeal carcinoma tissues using the Pearson correlation analysis. R = 0.7026. **G**. The expression of RAD51 was analyzed in NPC and NPE tissues using four NPC gene expression profile data (GSE12452, GSE53819, GSE64634, and GSE61218). Data were represented as mean ± SD. ns, not significant; *, *p* < 0.05; **, *p* < 0.01; ***, *p* < 0.001

H&E staining, in situ hybridization for circCDYL2 expression, and immunohistochemistry for RAD51 expression were performed in nude mice tissues (Fig. 5A). These investigations reaffirmed that circCDYL2 significantly elevated the expression of RAD51 protein (Fig. 5B), with a clear positive correlation between circCDYL2 and RAD51 expression in these xenograft tissues (Fig. 5C). Similarly, our findings were corroborated in archived clinical nasopharyngeal carcinoma tissue samples, where RAD51 exhibited high expression levels (Fig. 5D). Notably, a significant positive correlation was observed between elevated RAD51 expression, poorer patient prognosis (Fig. 5E), and circCDYL2 expression (Fig. 5F). Additionally, in gene expression profile data obtained from four sets of clinical nasopharyngeal carcinoma tissue samples downloaded from the GEO (Gene Expression Omnibus) database, RAD51 was markedly upregulated in nasopharyngeal carcinoma tissues compared to control tissues (Fig. 5G).

circCDYL2 originates from the CDYL2 (chromodomain Y like 2) gene. While the role of CDYL2 in malignant tumors is scarcely documented, CDYL (chromodomain Y like), a member of the CDYL family, has been reported to govern the post-translational modification of RPA1, thereby influencing HR repair [32]. We delved further into the question of whether circCDYL2 modulates RAD51 protein expression through CDYL2 mRNA or protein expression and whether this has any subsequent impact on DNA damage repair. Initially, we scrutinized the expression of CDYL2 in gene expression profile data from clinical nasopharyngeal carcinoma tissue samples and identified no significant disparity in CDYL2 expression between nasopharyngeal carcinoma and normal nasopharyngeal epithelial tissues (Supplemental Fig. 7A). Moreover, in nasopharyngeal carcinoma cells overexpressing or knocking down circCDYL2, RT-qPCR analysis revealed that circCDYL2 did not exert any influence on CDYL2 expression (Supplemental Fig. 7B). These findings suggest that circCDYL2 does not promote HR repair in nasopharyngeal carcinoma cells through regulating CDYL2 expression.

### circCDYL2 recruits EIF3D protein to facilitate translation initiation of RAD51

**Fig. 6:**
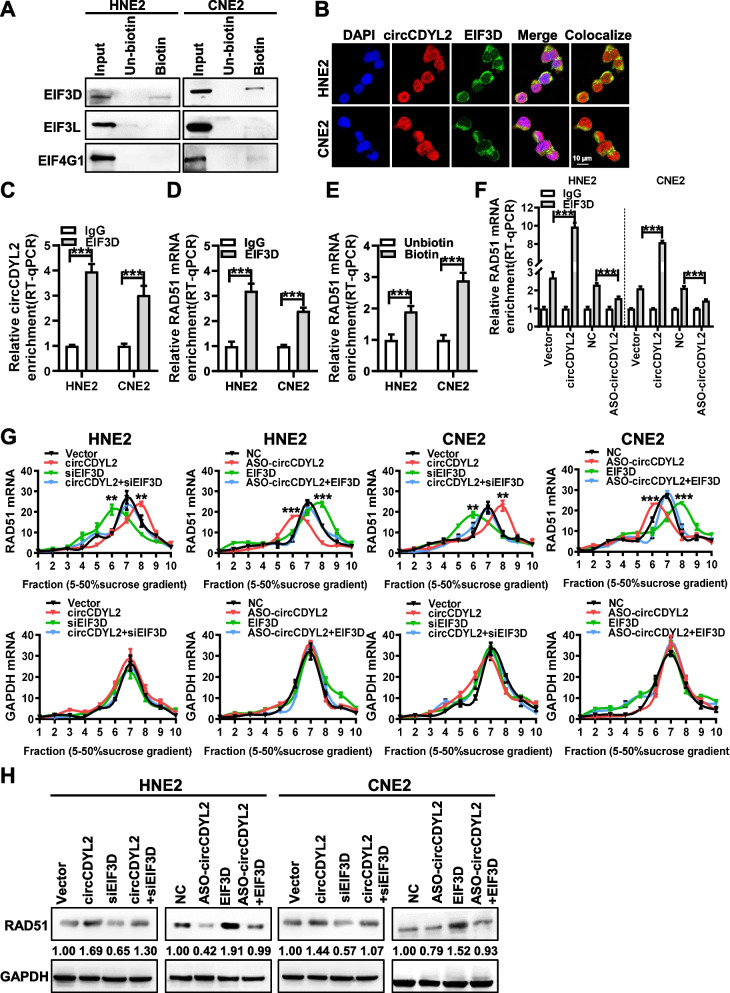
circCDYL2 recruits EIF3D protein to promote RAD51 translation initiation. **A**. RNA pulldown showed the interaction between circCDYL2 and EIF3D protein in HNE and CNE2 cells. **B**. Immunofluorescence demonstrated co-localization of circCDYL2 and EIF3D proteins in HNE and CNE2 cells. Blue, DAPI; Red, Dig-labeled circCDYL2; Green, anti-EIF3D antibody; Yellow, the co-localization of circCDYL2 and EIF3D protein. Scale bar = 10 μm. **C**. RNA immunoprecipitation (RIP) assay showed EIF3D proteins interacted with circCDYL2 in HNE2 and CNE2 cells. **D**. RIP experiments showed that EIF3D protein interacted with RAD51 mRNA in HNE2 and CNE2 cells. **E**. CircRIP experiments showed that circCDYL2 bound to RAD51 mRNA in HNE2 and CNE2 cells. **F**. RIP assays showed circCDYL2 enhanced the binding of EIF3D protein with RAD51 mRNA in NPC cells after overexpression or knockdown of circCDYL2. **G**. Polysome profiling was used to analyze the expression level of RAD51 mRNA in NPC cells after simultaneous knockdown of circCDYL2 with overexpression of EIF3D or overexpression of circCDYL2 with knockdown of EIF3D, respectively, using GAPDH mRNA as a control. **H**. Western blotting showed that EIF3D partially reverses the regulation of circCDYL2 on the expression of RAD51 protein in NPC cells after simultaneous knockdown of circCDYL2 with overexpression of EIF3D or overexpression of circCDYL2 with knockdown of EIF3D, respectively. Data were represented as mean ± SD. ns, not significant; *, *p* < 0.05; **, *p* < 0.01; ***, *p* < 0.001

To elucidate the mechanism by which circCDYL2 regulates RAD51 translation, we conducted RNA pulldown coupled with mass spectrometry analysis using the biotin-labeled circCDYL2 probes in CNE2 cells. This comprehensive approach identified a total of 162 candidate circCDYL2-binding proteins. The pathway enrichment analysis utilizing the Gene Ontology (GO) database revealed that the second-ranked pathway was translation regulation (Supplemental Table 5 and Supplemental Fig. 8A). This pathway encompassed several translation initiation factors, including EIF4G1, EIF3D, and EIF3L (Supplemental Table 6 and Supplemental Fig. 8B). We evaluated the top three translation initiation factors, and RNA pulldown confirmed the interaction between circCDYL2 and the translation initiation factor EIF3D protein (see Fig. 6A). Further, RNA FISH combined with immunofluorescence experiments demonstrated the co-localization of circCDYL2 and EIF3D protein in cells (Fig. 6B). RIP experiments provided additional evidence by confirming the binding of EIF3D protein to circCDYL2 (Fig. 6C). However, RT-qPCR and western blotting results showed that the overexpression or knockdown of circCDYL2 had no discernible effect on EIF3D expression (Supplemental Fig. 8C & D). Subsequent RIP experiments unveiled an interaction between EIF3D and RAD51 mRNA (Fig. 6D). As previous experiments had shown that circCDYL2 did not directly bind to the RAD51 protein (as in Fig. S4E), circRIP experiments provided further insights, demonstrating that circCDYL2 interacted with RAD51 mRNA (see Fig. 6E).

Following the overexpression or knockdown of circCDYL2 in nasopharyngeal carcinoma cells, RIP experiments revealed that the binding of EIF3D protein to RAD51 mRNA increased or decreased correspondingly (Fig. 6F). This observation suggests that circCDYL2 can facilitate the interaction between RAD51 mRNA and EIF3D protein. Polysome profiling assays further demonstrated that overexpressing or knocking down EIF3D (Supplemental Fig. 8E & F) in nasopharyngeal carcinoma cells could partially counteract circCDYL2’s influence on RAD51 translation (Fig. 6G). Western blotting results also confirmed that knockdown of EIF3D partially attenuated the heightened RAD51 expression induced by circCDYL2 overexpression, while overexpressing EIF3D partially reversed the reduced RAD51 expression resulting from circCDYL2 knockdown (Fig. 6H).

**Fig. 7:**
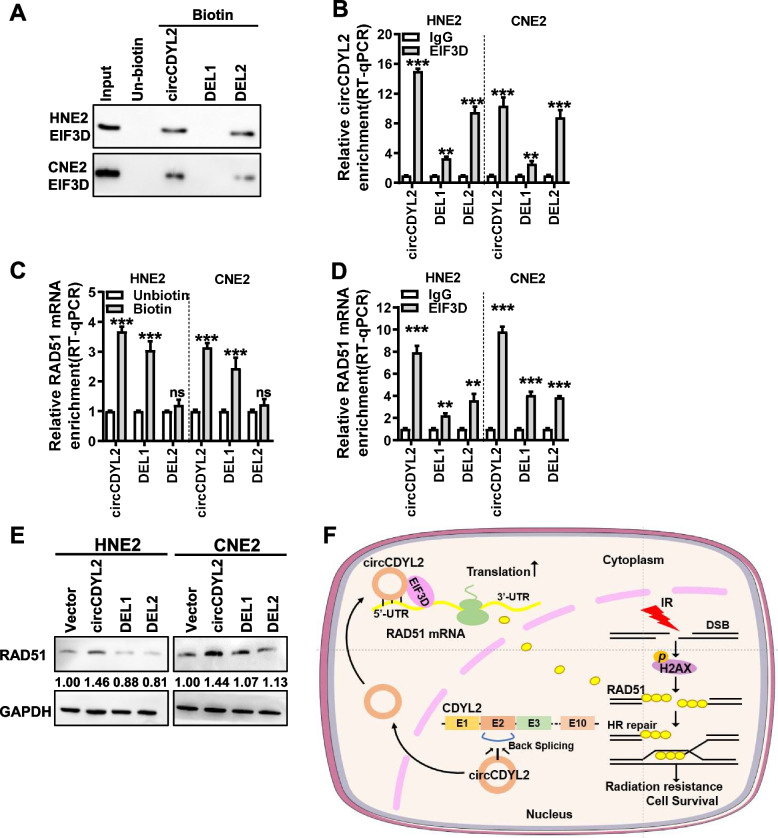
circCDYL2 serves as a scaffold to promote EIF3D protein binding to RAD51 mRNA. **A**. The binding site of circCDYL2 to EIF3D protein was determined by RNA pull down in HNE2 and CNE2 cells after overexpression of circCDYL2 or its deletion mutants (DEL1 and DEL2). **B**. The binding site of circCDYL2 to EIF3D protein was determined by RIP assay in HNE2 and CNE2 cells after overexpression of circCDYL2 or its deletion mutants (DEL1 and DEL2). **C**. The binding site of circCDYL2 to RAD51 mRNA was determined by circRIP in HNE2 and CNE2 cells after overexpression of circCDYL2 or its deletion mutants (DEL1 and DEL2). **D**. The binding between EIF3D and RAD51 mRNA was determined by RIP in HNE2 and CNE2 cells after overexpression of circCDYL2 or its deletion mutants (DEL1 and DEL2). **E**. The expression of RAD51 was detected by western blotting in HNE2 and CNE2 cells after overexpression of circCDYL2 or its deletion mutants (DEL1 and DEL2). **F**. Schematic diagram of circCDYL2 promoting radiotherapy resistance in nasopharyngeal carcinoma via the EIF3D/RAD51 axis. circCDYL2 is generated by reverse splicing of exon 2 of CDYL2 pre-mRNA. It interacts with EIF3D protein and RAD51 5′-UTR and acts as a scaffold to recruit EIF3D protein to bind with RAD51 mRNA, facilitating the translation of RAD51 and accelerating homologous recombination repair, which ultimately leads to radiotherapy resistance in nasopharyngeal carcinoma. Data were represented as mean ± SD. ns, not significant; *, *p* < 0.05; **, *p* < 0.01; ***, *p* < 0.001

The above experiments suggest that circCDYL2 may facilitate the initiation of RAD51 translation by recruiting EIF3D. To explore the relationships between circCDYL2, EIF3D, and RAD51 mRNA, we used the catRAPID software (http://service.tartaglialab.com/page/catrapid) to predict the binding site of circCDYL2 with EIF3D (as illustrated in Supplemental Fig. 9A). RNA hybrid (https://bibiserv.cebitec.uni-bielefeld.de/rnahybrid) prediction revealed that circCDYL2 exhibited reverse complementarity with a portion of the 5’-UTR of RAD51 mRNA (Supplemental Fig. 9B). In light of these bioinformatics analysis findings, we designed two circCDYL2 deletion mutants, DEL1 (1–20 nt) and DEL2 (310–329 nt), which lacked the potential binding site of circCDYL2 with EIF3D and the partial complementary pairing sequence of circCDYL2 with the 5’-UTR region of RAD51 mRNA respectively (Supplemental Fig. 9C & D). Utilizing the RNA fold software (http://rna.tbi.univie.ac.at/cgi-bin/RNAWebSuite/RNAfold.cgi), we obtained the secondary structure of circCDYL2 before and after mutation (Supplemental Fig. 9C). Upon successful overexpression of both wild-type and mutant circCDYL2 in cells (Supplemental Fig. 9E), RNA pulldown and RIP experiments unveiled that the 1–20 nt sequence of circCDYL2 (which is deleted in DEL1) plays a pivotal role in the interaction between circCDYL2 and EIF3D protein (Fig. 7A and B). Furthermore, the circRIP experiments indicated that the 310–329 nt sequence of circCDYL2 (which is deleted in DEL2) serves as the primary binding site for the interaction between circCDYL2 and RAD51 mRNA (Fig. 7C). Moreover, RIP experiments demonstrated that the overexpression of DEL1 and DEL2 attenuated the interaction between the EIF3D protein and RAD51 mRNA (Fig. 7D). Western blotting results further revealed that overexpressing DEL1 and DEL2 led to a partial reduction in RAD51 expression in comparison to overexpressing circCDYL2 (Fig. 7E). These findings provide additional support for the notion that circCDYL2 functions as a scaffold, promoting the interaction between EIF3D and RAD51 mRNA, and in turn, regulating RAD51 translation initiation.

## Discussion

Radiotherapy is currently one of the most commonly used treatments for tumors, alongside surgery and chemotherapy. Its fundamental mechanism involves the induction of irreparable DNA DSBs within cancer cells using ionizing radiation, ultimately leading to cell death [3, 26]. Tumor cells’ resistance to radiation represents a primary cause of treatment failure. The DNA damage repair system promptly activates a DNA damage response following a DNA double-strand break, recruiting numerous DNA damage repair proteins to the damaged sites and initiating a cascade reaction that facilitates DNA repair [33]. The main pathways for repairing DSBs include HR and NHEJ [34]. HR is predominantly mediated by RAD51. Upon DSB occurrence, the MRN (MRE11-RAD50-NBS1) sensor complex recognizes and binds to the structure, subsequently recruiting and activating ATM, leading to phosphorylation of H2AX (γH2AX) and amplifying the initial signal. Subsequently, numerous DNA damage repair proteins are recruited to the sites of DNA damage [35, 36]. CtIP, EXO1, and DNA2 exonuclease facilitate end resection to generate single-stranded DNA (ssDNA), which is then coated by RPA [37, 38]. The BRCA1-PALB2-BRCA2 complex recruits RAD51 to the damage site, displacing RPA and enabling RAD51 to utilize the undamaged homologous sister chromatid as a template for precise repair [25, 39]. In contrast, the NHEJ pathway is mediated by 53BP1, which inhibits DNA end resection, followed by direct ligation of the DNA double-strand break ends by the Ku80-Ku70 heterodimer and DNA-PKcs [40, 41]. In our study, we observed that circCDYL2 promotes HR repair rather than NHEJ, as evidenced by the DR-GFP and EJ5-GFP reporter systems. Examination of key molecules in the HR pathway unveiled that circCDYL2 enhances RAD51 expression. Further investigation revealed that circCDYL2 recruits EIF3D to promote RAD51 translation initiation, thereby fostering homologous recombination repair and contributing to radiation resistance in nasopharyngeal carcinoma.

Tumor cells evade radiation-induced cell death by proficiently repairing DNA double-strand breaks through the homologous recombination repair pathway, a primary contributor to radiotherapy resistance. Consequently, targeting the homologous recombination repair pathway emerges as a pivotal strategy for sensitizing tumor cells to radiotherapy. A comprehensive understanding of the regulatory mechanisms governing HR repair pathways and the identification of potential targets and molecular markers for radiotherapy sensitization hold significant clinical importance. These insights can lead to improved therapeutic outcomes, reduced tumor recurrence rates, and the ability to identify radiosensitive individuals, thereby mitigating the unnecessary side effects associated with excessive ionizing radiation.

circRNAs exert substantial influence over gene expression at multiple levels and have emerged as a cutting-edge and high-interest area in biomedical research. CircRNAs are widely distributed across various tissues and cells, exerting regulatory control over gene expression through diverse mechanisms. These mechanisms primarily include acting as miRNA sponges [21, 42] or binding to proteins [18, 43], thereby modulating downstream gene transcription or splicing processes [44, 45]. Additionally, circRNAs participate in epigenetic regulation [46], with certain instances of direct encoding of small peptides with biological functions [47, 48]. However, the exploration of circRNAs in the context of radiation resistance, particularly concerning DNA damage repair in nasopharyngeal carcinoma, remains limited. In this study, we have identified an elevated expression of circCDYL2 in nasopharyngeal carcinoma, and this upregulation is associated with an unfavorable prognosis for patients. By utilizing the DR-GFP and EJ5-GFP reporter systems, we have elucidated that both the overexpression and knockdown of circCDYL2 exert a significant impact on HR repair rather than NHEJ. Immunofluorescence assays have demonstrated that circCDYL2 promotes the formation of RAD51 foci, a crucial protein within the HR pathway, in nasopharyngeal carcinoma cells following radiation treatment. Our western blotting analysis has further validated that circCDYL2 elevates RAD51 expression. Subsequent investigations have unveiled that circCDYL2 actively enhances RAD51’s translation, thus promoting HR repair and contributing to radiotherapy resistance in nasopharyngeal carcinoma. It is worth noting that RAD51, being a pivotal component in HR repair, exhibits high expression in various solid tumors, and therefore, holds potential as a novel target for cancer treatment [49–51].

The role of circCDYL2 in malignant tumors and its underlying mechanisms remains to be fully elucidated. Previous research has confirmed that circCDYL2 promotes migration in colorectal cancer by binding to Ezrin and activating the AKT pathway [52]. Additionally, in breast cancer, circCDYL2 forms a complex with GRB7 and FAK, sustaining downstream signaling pathways of HER2 and contributing to trastuzumab resistance [53]. In our study, we provide the first evidence of circCDYL2’s involvement in promoting radiotherapy resistance in nasopharyngeal carcinoma. This unique mechanism centers around circCDYL2 acting as a scaffold molecule. Specifically, it binds to a portion of the 5’UTR of RAD51 mRNA through complementary base-pairing. Simultaneously, it recruits translation initiation factor EIF3D, to the 5’UTR of RAD51 mRNA, thus facilitating translation of RAD51 and enhancing RAD51 protein expression.

After mRNA maturation, the process of protein translation becomes a critical regulatory step. Protein translation encompasses three fundamental phases: translation initiation, elongation, and termination, all of which depend on the coordinated efforts of various translation-related factors [54, 55]. The translation initiation complex assumes a pivotal role in numerous tumor types [56–58]. Nonetheless, research on translation initiation in the context of radiation resistance is scarce. In our study, we employed RNA pull-down and mass spectrometry analysis to identify interactions between circCDYL2 and translation initiation complex-related proteins, including EIF3D. The EIF3 protein family, which constitutes the largest group of translation initiation factors in eukaryotes, comprising 13 family members, plays a central role in eukaryotic translation initiation [59]. Among these members, EIF3D holds particular significance. It has been previously reported that EIF3D directly interacts with RBMS1, with both factors binding to the 5’UTR and 3’UTR of SLC7A11, respectively. This interaction leads to the regulation of SLC7A11 expression by RBMS1 through EIF3D, consequently inhibiting ferroptosis and promoting the progression of lung cancer [60].

In summary, circCDYL2 has been a relatively unexplored molecule, and its function in tumors has remained elusive. Our study marks the first discovery of circCDYL2’s pronounced expression in nasopharyngeal carcinoma, where it correlates with a poor prognosis. In both in vivo and in vitro settings, we demonstrated that circCDYL2 actively promotes radiotherapy resistance in nasopharyngeal carcinoma cells. The mechanistic basis for this effect is rooted in circCDYL2’s role as a scaffold molecule. It facilitates the binding of EIF3D to RAD51 mRNA, thereby accelerating translation initiation and enhancing HR repair. This mechanism ultimately contributes to radiotherapy resistance in nasopharyngeal carcinoma (Fig. 7F). These findings underscore the critical importance of the circCDYL2/EIF3D/RAD51 axis in mediating radiotherapy resistance in nasopharyngeal carcinoma. Moreover, circCDYL2 has the potential to serve as a prognostic marker for adverse outcomes in nasopharyngeal carcinoma. Targeting circCDYL2 and RAD51 could represent promising therapeutic strategies for sensitizing nasopharyngeal carcinoma to radiotherapy.

### Supplementary Information

**Additional file 1 Supplemental Fig. 1**. circCDYL2 promotes radiotherapy resistance in nasopharyngeal carcinoma cells. A. Clonogenic assays demonstrate that CNE2-IR cells exhibited higher radiotherapy resistance, compared with CNE2 cells, after exposure to 2 Gy radiation. B. The expression of circCDYL2 was detected by RT-qPCR in CNE2 and CNE2-IR cells. C. Transfection efficiency was evaluated using RT-qPCR in HNE2 and CNE2 cells after overexpression or knockdown of circCDYL2. D. The levels of γ-H2AX expression were detected by immunofluorescence in HNE2 and CNE2 cells after overexpression or knockdown of circCDYL2 with 6 Gy irradiation for 2, 6, 12, and 24 hours. The results showed that overexpression of circCDYL2 reduced the accumulation of radiation-induced foci (IRIF) of γ-H2AX post-irradiation, while circCDYL2 knockdown had the opposite effect. Scale bar = 10 μm. The right graph represents the number of radiation-induced foci (IRIF) in 30 cells. Data were represented as mean ± SD. ns, not significant; *, *p* < 0.05; **, *p* < 0.01; ***, *p* < 0.001. **Supplemental Fig. 2**. circCDYL2 promotes homologous recombination repair. A. Schematic diagram of the HR and NHEJ repair pathways. B. Efficiency of BRCA1 and 53BP1 knockdown was determined by RT-qPCR and western blotting in DR-GFP-U2OS and EJ5-GFP-U2OS cells. C. The impact of overexpression or knockdown of circCDYL2 on HR and NHEJ repair efficiency was detected by Flow cytometry. Data were represented as mean ± SD. ns, not significant; *, *p* < 0.05; **, *p* < 0.01; ***, *p* < 0.001. **Supplemental Fig. 3**. circCDYL2 does not influence the expression of BRCA1, 53BP1, or KU70. A. Immunofluorescence showed that circCDYL2 did not affect the accumulation of BRCA1 IRIF in NPC cells after overexpression or knockdown of circCDYL2. Scale bar = 10 μm. On the right, quantification of IRIF per 30 cells was presented. B. Immunofluorescence showed that circCDYL2 did not affect the accumulation of RPA1 IRIF in NPC cells after overexpression or knockdown of circCDYL2. Scale bar = 10 μm. On the right, quantification of IRIF per 30 cells was presented. C. The expression of DNA repair-related proteins BRCA1, RPA1, and Ku70 was detected by western blotting in NPC cells after overexpression or knockdown of circCDYL2. The results showed that circCDYL2 did not affect their expression. Data were represented as mean ± SD. ns, not significant; *, *p* < 0.05; **, *p* < 0.01; ***, *p* < 0.001. **Supplemental Fig. 4**. circCDYL2 does not influence the stability of RAD51. A. RT-qPCR showed that circCDYL2 did not affect the mRNA level of RAD51 in NPC cells after overexpression or knockdown of circCDYL2. B. RT-qPCR showed that circCDYL2 did not affect the stability of RAD51 mRNA in NPC cells after overexpression or knockdown of circCDYL2. Cells were treated with actinomycin D for 0, 0.5, 1, or 2 hours, respectively. C, D. Western blotting demonstrated that circCDYL2 did not affect the half-life and ubiquitination degradation of RAD51 in NPC cells after overexpression or knockdown of circCDYL2. Cells were treated with cycloheximide (CHX) (50 μg/ml) or MG132 (20 μM) for 0, 6, 12, or 24 hours, respectively. E. RNA pull-down revealed that circCDYL2 did not interact with RAD51 protein. Data were represented as mean ± SD. ns, not significant; *, *p* < 0.05; **, *p* < 0.01; ***, *p* < 0.001. **Supplemental Fig. 5**. circCDYL2 enhances RAD51 translation to promote DNA homologous recombination repair in nasopharyngeal carcinoma. A. Polysome profiling demonstrated that GAPDH mRNA expression on polysomes was not affected by circCDYL2 in NPC cells after overexpression or knockdown of circCDYL2. Data were represented as mean ± SD. B. Flow cytometry showed that RAD51 could partially reverse the effect of circCDYL2 on homologous recombination repair in DR-GFP U2OS cells after simultaneous knockdown of circCDYL2 with overexpression of RAD51 or overexpression of circCDYL2 combined with knockdown of RAD51, respectively. Each group was co-transfected with the HA-Isce1 overexpression plasmid. Data were represented as mean ± SD. ns, not significant; *, *p* < 0.05; **, *p* < 0.01; ***, *p* < 0.001. **Supplemental Fig. 6**. circCDYL2 promotes radiotherapy resistance in nasopharyngeal carcinoma by upregulation of RAD51. A. Clonogenic assays showed that RAD51 partially reversed the effect of circCDYL2 on CNE2 cell survival post-irradiation after simultaneous overexpression of circCDYL2 combined with knockdown of RAD51 or knockdown of circCDYL2 with overexpression of RAD51, respectively. These cells were exposed to 0, 2, 4, 6, and 8 Gy X-ray irradiation, respectively. B. Immunofluorescence showed that RAD51 could partially reverse the regulation of γ-H2AX radiation-induced foci (IRIF) by circCDYL2 in CNE2 cells after simultaneous overexpression of circCDYL2 combined with knockdown of RAD51 or knockdown of circCDYL2 with overexpression of RAD51, respectively. These cells were irradiated with 6 Gy X-rays for 6 hours. Scale bar = 10 μm. C. Western blotting demonstrated that RAD51 could partially reverse the regulation of γ-H2AX expression levels by circCDYL2 in NPC cells after simultaneous overexpression of circCDYL2 combined with knockdown of RAD51 or knockdown of circCDYL2 with overexpression of RAD51, respectively. These cells were irradiated with 6 Gy X-rays for 0, 2, 6, 12, or 24 hours, respectively. D. Comet assays showed that RAD51 mediated the effect of circCDYL2 on DNA damage repair in CNE2 cells after simultaneous overexpression of circCDYL2 combined with knockdown of RAD51 or knockdown of circCDYL2 with overexpression of RAD51, respectively. These cells were irradiated with 6 Gy X-rays, and. Scale bar = 20 μm. Data were represented as mean ± SD. ns, not significant; *, *p* < 0.05; **, *p* < 0.01; ***, *p* < 0.001. **Supplemental Fig. 7**. circCDYL2 does not influence the expression of CDYL2. A. The expression of CDYL2 was analyzed in NPC GEO datasets (GSE12452, GSE53819, GSE64634, and GSE61218). B. The expression of CDYL2 mRNA was detected by RT-qPCR in HNE2 and CNE2 cells after overexpression or knockdown of circCDYL2. Data were represented as mean ± SD. ns, not significant; *, *p* < 0.05; **, *p* < 0.01; ***, *p* < 0.001. **Supplemental Fig. 8**. circCDYL2 interacts with EIF3D but does not regulate its expression. A. The biotin-labeled circCDYL2 probe was used for RNA pulldown, and LC-MS/MS was used to identify proteins interacting with circCDYL2 in CNE2 cells. Pathways regulated by circCDYL2 were enriched using the GO database. B. Translation initiation factors, including EIF4G1, EIF3D, EIF3L, EIF2A, EIF2AK2, EIF4G2, and EIF3C, were ranked based on their score values from mass spectrometry data. C. RT-qPCR showed that circCDYL2 did not regulate the expression of EIF3D mRNA in HNE2 and CNE2 cells after overexpression or knockdown of circCDYL2. D. Western blotting demonstrated that circCDYL2 did not regulate the expression of EIF3D protein in HNE2 and CNE2 cells after overexpression or knockdown of circCDYL2. E. The transfection efficiency of EIF3D was detected by RT-qPCR in NPC cells after overexpression or knockdown of EIF3D. F. The transfection efficiency of EIF3D was detected by western blotting in NPC cells after overexpression or knockdown of EIF3D. Data were represented as mean ± SD. ns, not significant; *, *p* < 0.05; **, *p* < 0.01; ***, *p* < 0.001. **Supplemental Fig. 9**. circCDYL2 interacts with both EIF3D protein and RAD51 mRNA. A. The binding site of circCDYL2 to EIF3D protein was predicted by the catRAPID software. B. The binding site of circCDYL2 to RAD51 mRNA was predicted by the RNA hybrid software. C. Two mutants of circCDYL2 (DEL1 and DEL2) were designed according to the secondary structure of circCDYL2 indicated by the RNA fold software. D. Schematic diagram of the deleted regions of the circCDYL2 mutants. E. The transfection efficiency of circCDYL2 was examined by qRT-PCR in HNE2 and CNE2 cells after overexpression of circCDYL2 or its deletion mutants (DEL1 and DEL2). Data were represented as mean ± SD. ns, not significant; *, *p* < 0.05; **, *p* < 0.01; ***, *p* < 0.001. **Supplemental Table 1**. Clinicopathological data of 45 NPC and 23 NPE tissues measured by RT-qPCR. **Supplemental Table 2**. Clinicopathological data of 203 paraffin-embedded NPC tissues and 56 non-neoplastic nasopharyngeal epithelial tissues for in situ hybridization. **Supplemental Table 3**. Primers, probes, and siRNA used in this study. **Supplemental Table 4**. Antibodies used in this study. **Supplemental Table 5**. Potential circCDYL2 interacting proteins in HNE2 cells using the LC-MS/MS method after pulldown by the biotin-labeled circCDYL2 probe. **Supplemental Table 6**. Translation initiation factors according to the LC-MS/MS data.

## Data Availability

All data that support the findings of this study are available from the corresponding authors upon reasonable request.

## Abbreviations

NPC: Nasopharyngeal carcinoma
miRNA: MicroRNA
lncRNA: Long non-coding RNA
circRNA: Circular RNA
FISH: Fluorescence in situ hybridization
ISH: In situ hybridization
RIP: RNA immunoprecipitation
CDYL2: Chromodomain Y like 2
DSBs: Double-strand breaks
HR: Homologous recombination
NHEJ: Non-homologous end joining
BRCA1: Breast cancer susceptibility gene 1
53BP1: Tumor protein p53 binding protein 1
RPA1: Replication Protein A1
CHX: Cycloheximide
GEO: Gene Expression Omnibus
GO: Gene Ontology
EIF3D: Eukaryotic translation initiation factor 3 subunit
ATM: Ataxia telangiectasia-mutated kinase
BRCA1: Breast cancer susceptibility gene 1
PALB2: Partner and localizer of BRCA2
BRCA2: Breast cancer susceptibility gene 2
