# Supplementary material for: CircCDYL2 bolsters radiotherapy resistance in nasopharyngeal carcinoma by promoting RAD51 translation initiation for enhanced homologous recombination repair
Source: J Exp Clin Cancer Res. 2024 Apr 23;43:122. doi: 10.1186/s13046-024-03049-0 (PMC11036759; doi:10.1186/s13046-024-03049-0)
Supplement: Supplementary file 1 — Additional file 1 Supplemental Fig. 1. circCDYL2 promotes radiotherapy resistance in nasopharyngeal carcinoma cells. A. Clonogenic assays demonstrate that CNE2-IR cells exhibited higher radiotherapy resistance, compared with CNE2 cells, after exposure to 2 Gy radiation. B. The expression of circCDYL2 was detected by RT-qPCR in CNE2 and CNE2-IR cells. C. Transfection efficiency was evaluated using RT-qPCR in HNE2 and CNE2 cells after overexpression or knockdown of circCDYL2. D. The levels of γ-H2AX expression were detected by immunofluorescence in HNE2 and CNE2 cells after overexpression or knockdown of circCDYL2 with 6 Gy irradiation for 2, 6, 12, and 24 hours. The results showed that overexpression of circCDYL2 reduced the accumulation of radiation-induced foci (IRIF) of γ-H2AX post-irradiation, while circCDYL2 knockdown had the opposite effect. Scale bar = 10 μm. The right graph represents the number of radiation-induced foci (IRIF) in 30 cells. Data were represented as mean ± SD. ns, not significant; *, p < 0.05; **, p < 0.01; ***, p < 0.001. Supplemental Fig. 2. circCDYL2 promotes homologous recombination repair. A. Schematic diagram of the HR and NHEJ repair pathways. B. Efficiency of BRCA1 and 53BP1 knockdown was determined by RT-qPCR and western blotting in DR-GFP-U2OS and EJ5-GFP-U2OS cells. C. The impact of overexpression or knockdown of circCDYL2 on HR and NHEJ repair efficiency was detected by Flow cytometry. Data were represented as mean ± SD. ns, not significant; *, p < 0.05; **, p < 0.01; ***, p < 0.001. Supplemental Fig. 3. circCDYL2 does not influence the expression of BRCA1, 53BP1, or KU70. A. Immunofluorescence showed that circCDYL2 did not affect the accumulation of BRCA1 IRIF in NPC cells after overexpression or knockdown of circCDYL2. Scale bar = 10 μm. On the right, quantification of IRIF per 30 cells was presented. B. Immunofluorescence showed that circCDYL2 did not affect the accumulation of RPA1 IRIF in NPC cells after overexpression or knoc [file 13046_2024_3049_MOESM1_ESM.pdf]

Figure S1

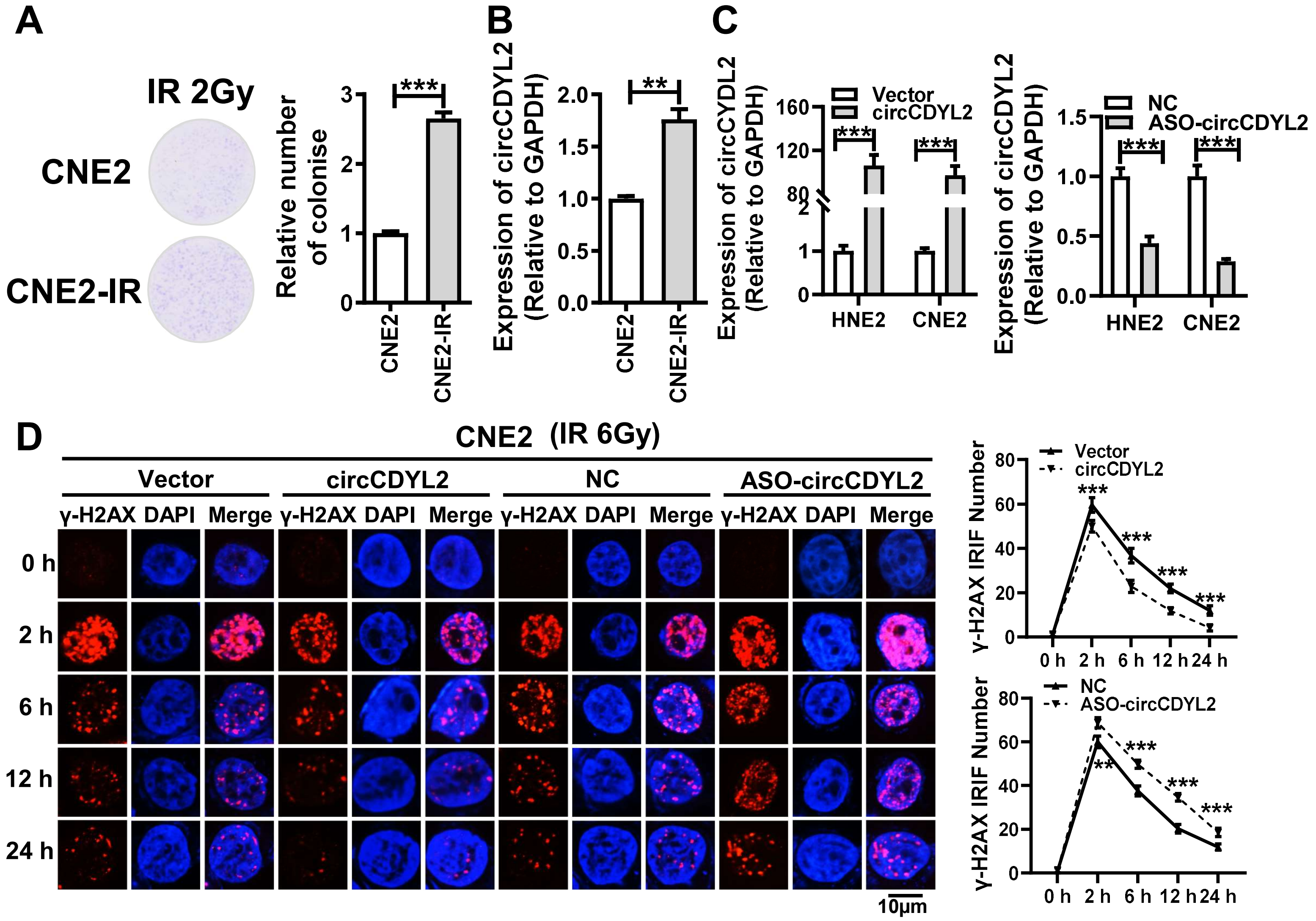
A
IR 2Gy
CNE2
CNE2-IR
Relative number of colonise
***
CNE2
CNE2-IR
B
Expression of circCDYL2 (Relative to GAPDH)
**
CNE2
CNE2-IR
C
Expression of circCYDL2 (Relative to GAPDH)
Vector
circCDYL2
***
***
HNE2
CNE2
Expression of circCDYL2 (Relative to GAPDH)
NC
ASO-circCDYL2
***
***
HNE2
CNE2
D
CNE2 (IR 6Gy)
Vector
circCDYL2
NC
ASO-circCDYL2
γ-H2AX DAPI Merge
0 h
2 h
6 h
12 h
24 h
10μm
γ-H2AX IRIF Number
Vector
circCDYL2
***
***
***
***
0 h 2 h 6 h 12 h 24 h
γ-H2AX IRIF Number
NC
ASO-circCDYL2
**
***
***
***
0 h 2 h 6 h 12 h 24 h

Supplemental Figure 1. circCDYL2 promotes radiotherapy resistance in nasopharyngeal carcinoma cells.

A. Clonogenic assays demonstrate that CNE2-IR cells exhibited higher radiotherapy resistance, compared with CNE2 cells, after exposure to 2 Gy radiation.

B. The expression of circCDYL2 was detected by RT-qPCR in CNE2 and CNE2-IR cells.

C. Transfection efficiency was evaluated using RT-qPCR in HNE2 and CNE2 cells after overexpression or knockdown of circCDYL2.

D. The levels of γ-H2AX expression were detected by immunofluorescence in HNE2 and CNE2 cells after overexpression or knockdown of circCDYL2 with 6 Gy irradiation for 2, 6, 12, and 24 hours. The results showed that overexpression of circCDYL2 reduced the accumulation of radiation-induced foci (IRIF) of γ-H2AX post-irradiation, while circCDYL2 knockdown had the opposite effect. Scale bar = 10 μm. The right graph represents the number of radiation-induced foci (IRIF) in 30 cells. Data were represented as mean ± SD. ns, not significant; *, $p < 0.05$; **, $p < 0.01$; ***, $p < 0.001$.

Figure S2

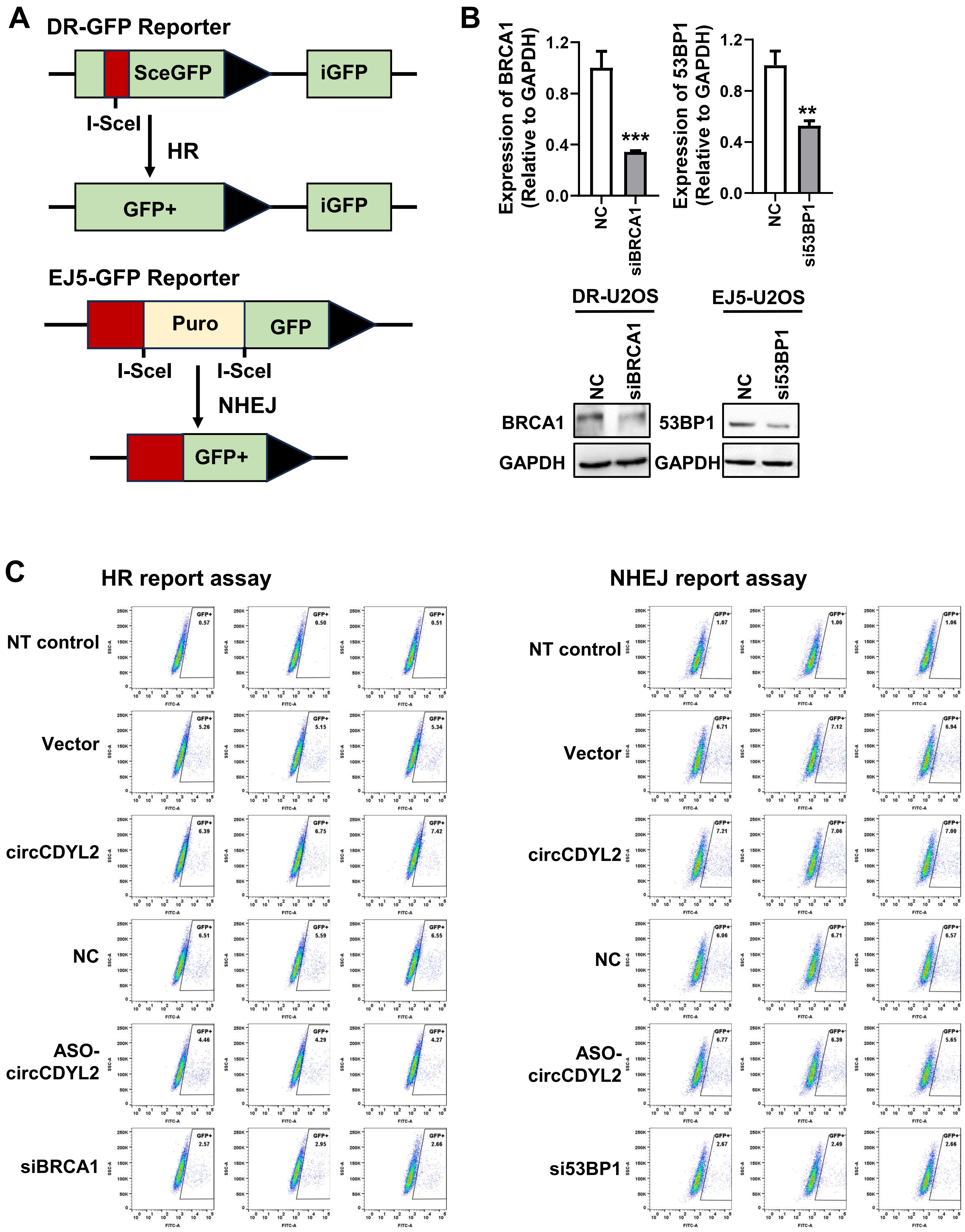
A
DR-GFP Reporter
SceGFP
iGFP
I-SceI
HR
GFP+
iGFP
EJ5-GFP Reporter
Puro
GFP
I-SceI
I-SceI
NHEJ
GFP+
B
Expression of BRCA1 (Relative to GAPDH)
NC
siBRCA1
***
Expression of 53BP1 (Relative to GAPDH)
NC
si53BP1
**
DR-U2OS
EJ5-U2OS
BRCA1
GAPDH
53BP1
GAPDH
C
HR report assay
NHEJ report assay
NT control
Vector
circCDYL2
NC
ASO-circCDYL2
siBRCA1
si53BP1

Supplemental Figure 2. circCDYL2 promotes homologous recombination repair.

A. Schematic diagram of the HR and NHEJ repair pathways.

B. Efficiency of BRCA1 and 53BP1 knockdown was determined by RT-qPCR and western blotting in DR-GFP-U2OS and EJ5-GFP-U2OS cells.

C. The impact of overexpression or knockdown of circCDYL2 on HR and NHEJ repair efficiency was detected by Flow cytometry.

Data were represented as mean ± SD. ns, not significant; *, $p < 0.05$; **, $p < 0.01$; ***, $p < 0.001$.

Figure S3

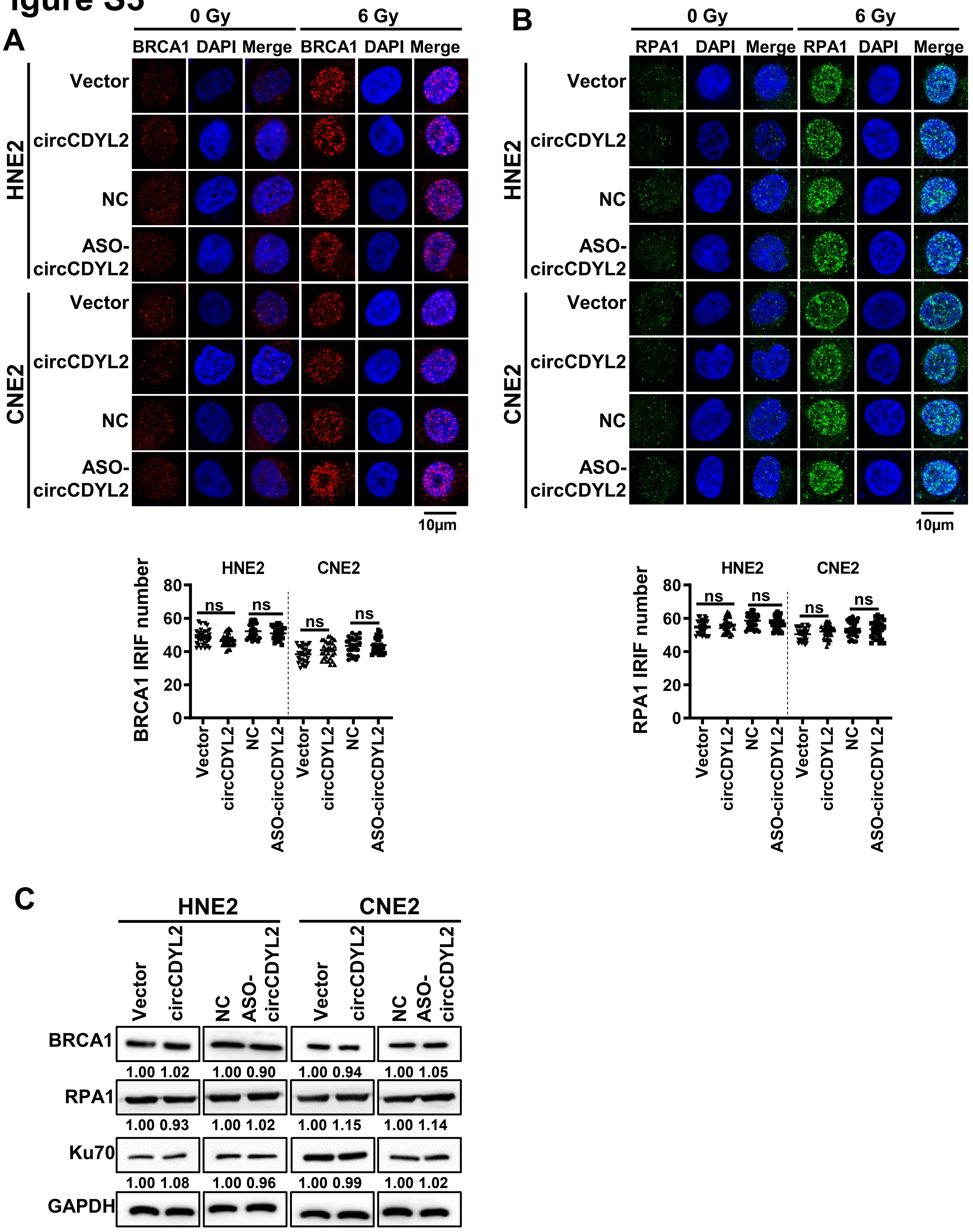
A
0 Gy
6 Gy
BRCA1 DAPI Merge BRCA1 DAPI Merge
HNE2
CNE2
Vector
circCDYL2
NC
ASO-circCDYL2
10μm
B
0 Gy
6 Gy
RPA1 DAPI Merge RPA1 DAPI Merge
10μm
BRCA1 IRIF number
RPA1 IRIF number
ns
C
HNE2
CNE2
BRCA1
1.00 1.02 1.00 0.90 1.00 0.94 1.00 1.05
RPA1
1.00 0.93 1.00 1.02 1.00 1.15 1.00 1.14
Ku70
1.00 1.08 1.00 0.96 1.00 0.99 1.00 1.02
GAPDH

Supplemental Figure 3. circCDYL2 does not influence the expression of BRCA1, 53BP1, or KU70.

A. Immunofluorescence showed that circCDYL2 did not affect the accumulation of BRCA1 IRIF in NPC cells after overexpression or knockdown of circCDYL2. Scale bar = 10 μm. On the right, quantification of IRIF per 30 cells was presented.

B. Immunofluorescence showed that circCDYL2 did not affect the accumulation of RPA1 IRIF in NPC cells after overexpression or knockdown of circCDYL2. Scale bar = 10 μm. On the right, quantification of IRIF per 30 cells was presented.

C. The expression of DNA repair-related proteins BRCA1, RPA1, and Ku70 was detected by western blotting in NPC cells after overexpression or knockdown of circCDYL2. The results showed that circCDYL2 did not affect their expression.

Data were represented as mean ± SD. ns, not significant; *, $p < 0.05$; **, $p < 0.01$; ***, $p < 0.001$.

Figure S4

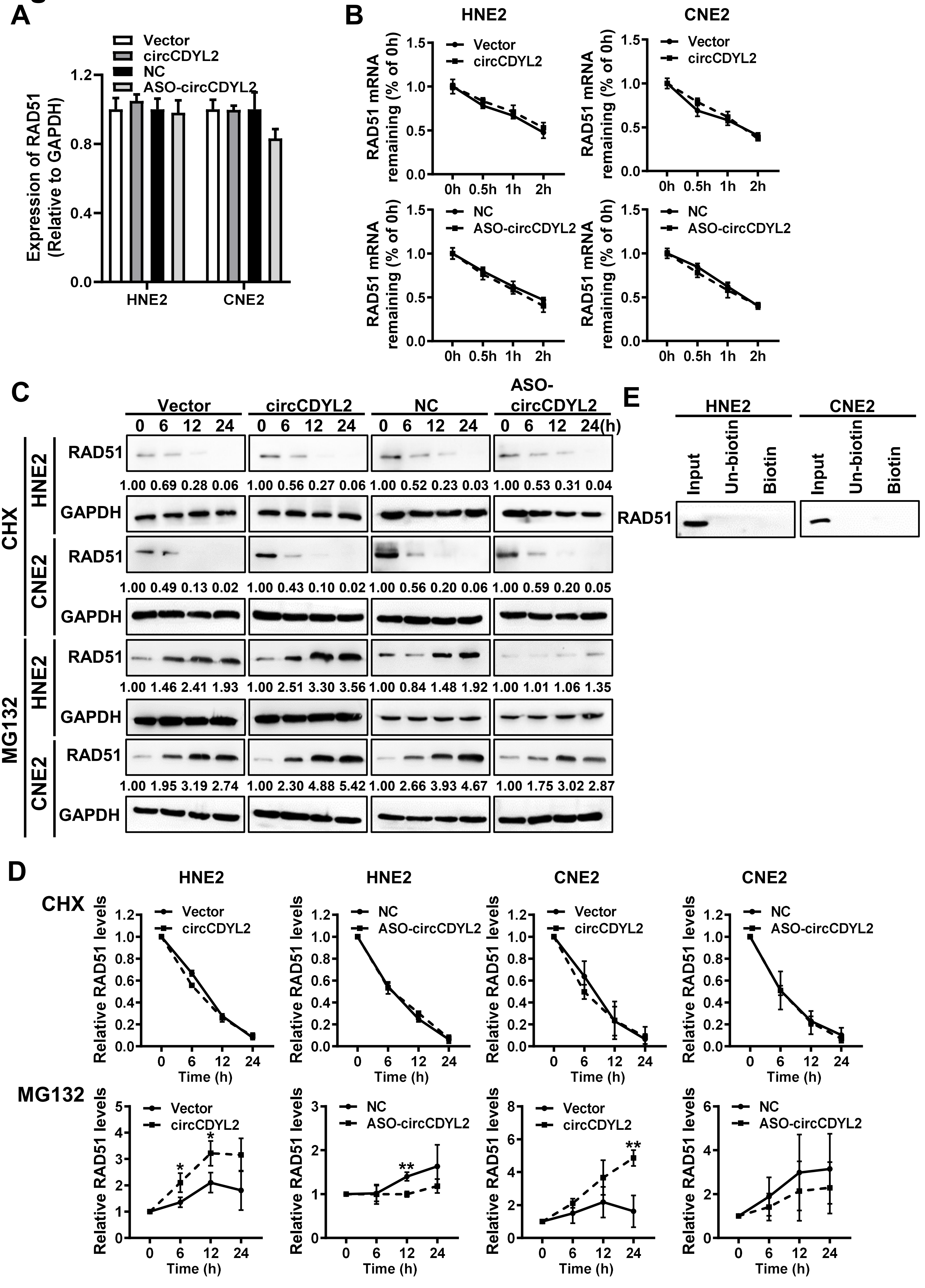
A
Vector
circCDYL2
NC
ASO-circCDYL2
Expression of RAD51 (Relative to GAPDH)
HNE2
CNE2
B
HNE2
CNE2
RAD51 mRNA remaining (% of 0h)
0h 0.5h 1h 2h
C
Vector
circCDYL2
NC
ASO-circCDYL2
0 6 12 24 (h)
CHX
MG132
HNE2
CNE2
RAD51
GAPDH
1.00 0.69 0.28 0.06 1.00 0.56 0.27 0.06 1.00 0.52 0.23 0.03 1.00 0.53 0.31 0.04
1.00 0.49 0.13 0.02 1.00 0.43 0.10 0.02 1.00 0.56 0.20 0.06 1.00 0.59 0.20 0.05
1.00 1.46 2.41 1.93 1.00 2.51 3.30 3.56 1.00 0.84 1.48 1.92 1.00 1.01 1.06 1.35
1.00 1.95 3.19 2.74 1.00 2.30 4.88 5.42 1.00 2.66 3.93 4.67 1.00 1.75 3.02 2.87
E
HNE2
CNE2
Input
Un-biotin
Biotin
RAD51
D
HNE2
CNE2
CHX
MG132
Relative RAD51 levels
Time (h)

Supplemental Figure 4. circCDYL2 does not influence the stability of RAD51.

A. RT-qPCR showed that circCDYL2 did not affect the mRNA level of RAD51 in NPC cells after overexpression or knockdown of circCDYL2.

B. RT-qPCR showed that circCDYL2 did not affect the stability of RAD51 mRNA in NPC cells after overexpression or knockdown of circCDYL2. Cells were treated with actinomycin D for 0, 0.5, 1, or 2 hours, respectively.

C, D. Western blotting demonstrated that circCDYL2 did not affect the half-life and ubiquitination degradation of RAD51 in NPC cells after overexpression or knockdown of circCDYL2. Cells were treated with cycloheximide (CHX) (50 μg/ml) or MG132 (20 μM) for 0, 6, 12, or 24 hours, respectively.

E. RNA pull-down revealed that circCDYL2 did not interact with RAD51 protein.

Data were represented as mean ± SD. ns, not significant; *, $p < 0.05$; **, $p < 0.01$; ***, $p < 0.001$.

Figure S5

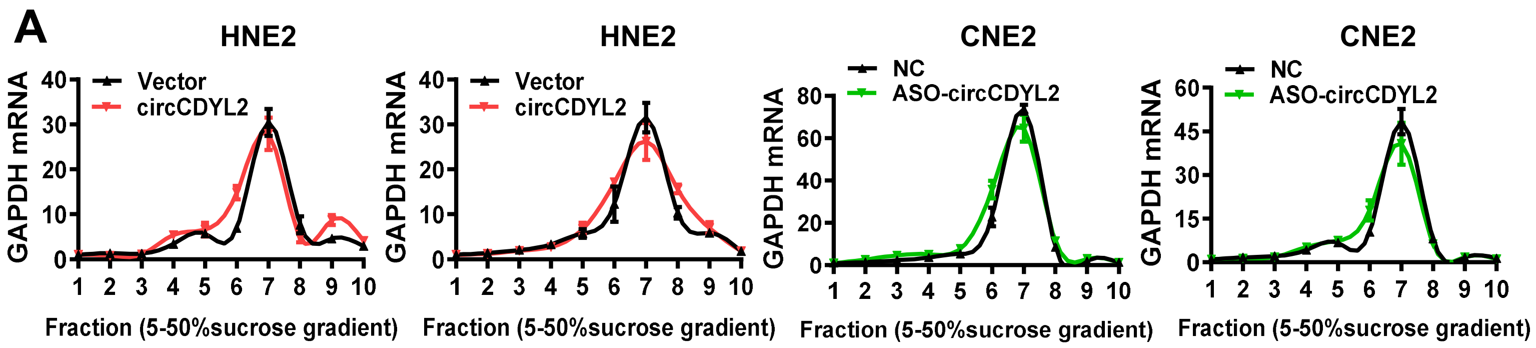

A
HNE2
GAPDH mRNA
Vector
circCDYL2
Fraction (5-50%sucrose gradient)
HNE2
Vector
circCDYL2
CNE2
NC
ASO-circCDYL2
CNE2
NC
ASO-circCDYL2

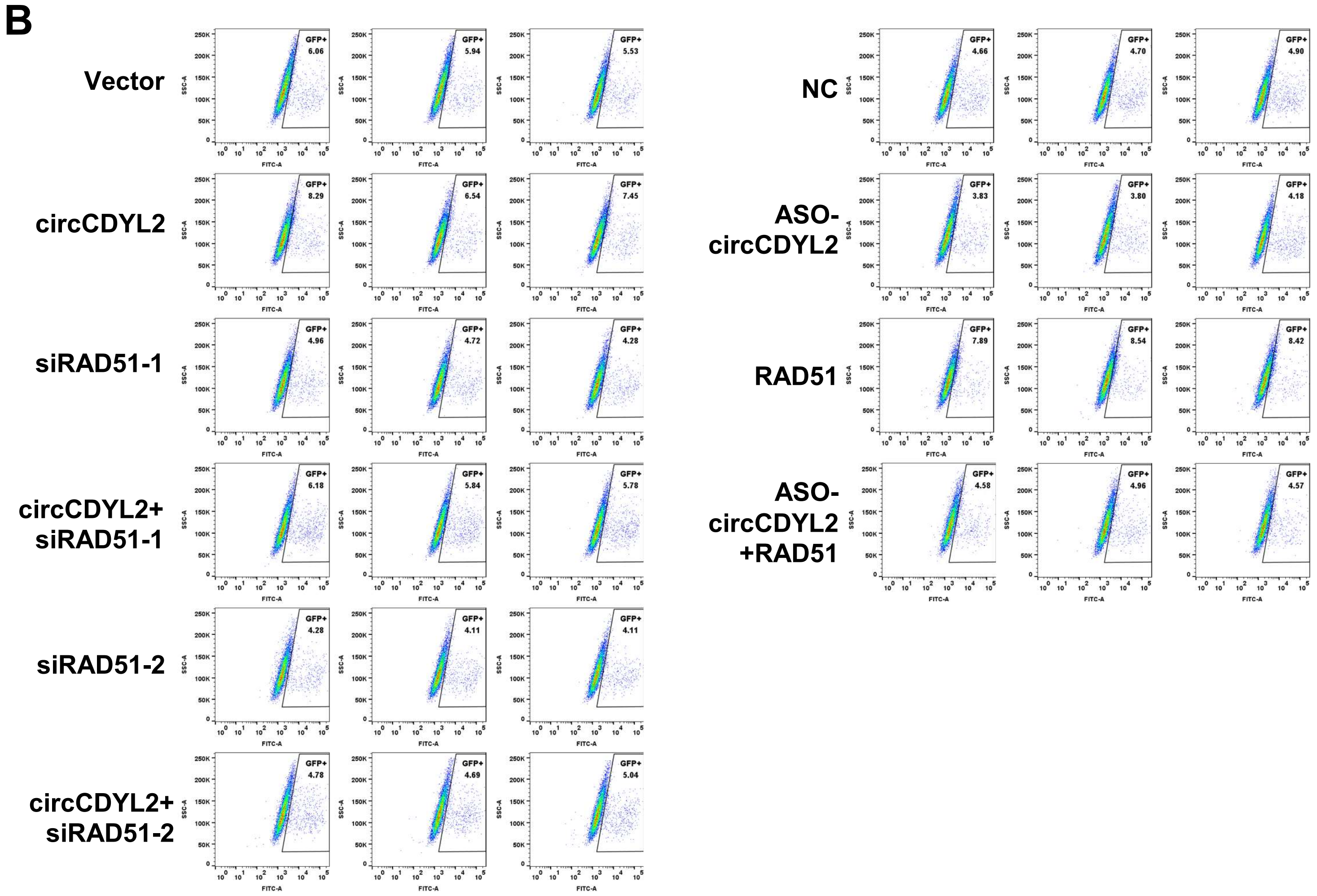

B
Vector
circCDYL2
siRAD51-1
circCDYL2+
siRAD51-1
siRAD51-2
circCDYL2+
siRAD51-2
NC
ASO-
circCDYL2
RAD51
ASO-
circCDYL2
+RAD51

Supplemental Figure 5. circCDYL2 enhances RAD51 translation to promote DNA homologous recombination repair in nasopharyngeal carcinoma.

A. Polysome profiling demonstrated that GAPDH mRNA expression on polysomes was not affected by circCDYL2 in NPC cells after overexpression or knockdown of circCDYL2. Data were represented as mean ± SD.

B. Flow cytometry showed that RAD51 could partially reverse the effect of circCDYL2 on homologous recombination repair in DR-GFP U2OS cells after simultaneous knockdown of circCDYL2 with overexpression of RAD51 or overexpression of circCDYL2 combined with knockdown of RAD51, respectively. Each group was co-transfected with the HA-Isce1 overexpression plasmid.

Data were represented as mean ± SD. ns, not significant; *, $p < 0.05$; **, $p < 0.01$; ***, $p < 0.001$.

Figure S6

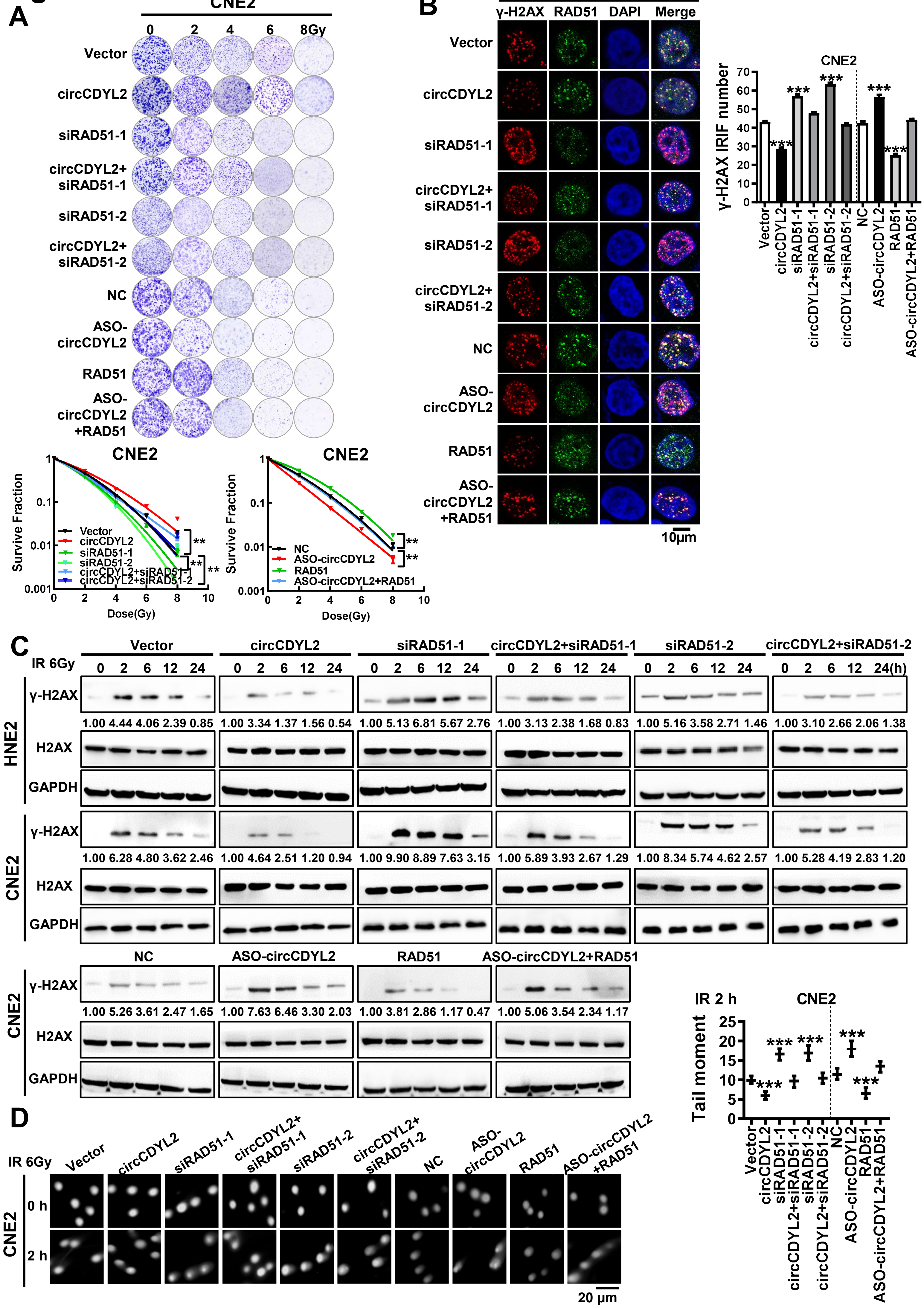
A
CNE2
0
2
4
6
8Gy
Vector
circCDYL2
siRAD51-1
circCDYL2+ siRAD51-1
siRAD51-2
circCDYL2+ siRAD51-2
NC
ASO-circCDYL2
RAD51
ASO-circCDYL2 +RAD51
CNE2
Survive Fraction
Dose(Gy)
Vector
circCDYL2
siRAD51-1
siRAD51-2
circCDYL2+siRAD51-1
circCDYL2+siRAD51-2
**
NC
ASO-circCDYL2
RAD51
ASO-circCDYL2+RAD51
B
CNE2
γ-H2AX
RAD51
DAPI
Merge
10μm
γ-H2AX IRIF number
***
C
IR 6Gy
Vector
circCDYL2
siRAD51-1
circCDYL2+siRAD51-1
siRAD51-2
circCDYL2+siRAD51-2
0 2 6 12 24(h)
HNE2
γ-H2AX
1.00 4.44 4.06 2.39 0.85
1.00 3.34 1.37 1.56 0.54
1.00 5.13 6.81 5.67 2.76
1.00 3.13 2.38 1.68 0.83
1.00 5.16 3.58 2.71 1.46
1.00 3.10 2.66 2.06 1.38
H2AX
GAPDH
CNE2
γ-H2AX
1.00 6.28 4.80 3.62 2.46
1.00 4.64 2.51 1.20 0.94
1.00 9.90 8.89 7.63 3.15
1.00 5.89 3.93 2.67 1.29
1.00 8.34 5.74 4.62 2.57
1.00 5.28 4.19 2.83 1.20
H2AX
GAPDH
NC
ASO-circCDYL2
RAD51
ASO-circCDYL2+RAD51
CNE2
γ-H2AX
1.00 5.26 3.61 2.47 1.65
1.00 7.63 6.46 3.30 2.03
1.00 3.81 2.86 1.17 0.47
1.00 5.06 3.54 2.34 1.17
H2AX
GAPDH
IR 2 h
CNE2
Tail moment
D
IR 6Gy
CNE2
0 h
2 h
20 μm

Supplemental Figure 6. circCDYL2 promotes radiotherapy resistance in nasopharyngeal carcinoma by upregulation of RAD51.

A. Clonogenic assays showed that RAD51 partially reversed the effect of circCDYL2 on CNE2 cell survival post-irradiation after simultaneous overexpression of circCDYL2 combined with knockdown of RAD51 or knockdown of circCDYL2 with overexpression of RAD51, respectively. These cells were exposed to 0, 2, 4, 6, and 8 Gy X-ray irradiation, respectively.

B. Immunofluorescence showed that RAD51 could partially reverse the regulation of γ-H2AX radiation-induced foci (IRIF) by circCDYL2 in CNE2 cells after simultaneous overexpression of circCDYL2 combined with knockdown of RAD51 or knockdown of circCDYL2 with overexpression of RAD51, respectively. These cells were irradiated with 6 Gy X-rays for 6 hours. Scale bar = 10 μm.

C. Western blotting demonstrated that RAD51 could partially reverse the regulation of γ-H2AX expression levels by circCDYL2 in NPC cells after simultaneous overexpression of circCDYL2 combined with knockdown of RAD51 or knockdown of circCDYL2 with overexpression of RAD51, respectively. These cells were irradiated with 6 Gy X-rays for 0, 2, 6, 12, or 24 hours, respectively.

D. Comet assays showed that RAD51 mediated the effect of circCDYL2 on DNA damage repair in CNE2 cells after simultaneous overexpression of circCDYL2 combined with knockdown of RAD51 or knockdown of circCDYL2 with overexpression of RAD51, respectively. These cells were irradiated with 6 Gy X-rays, and. Scale bar = 20 μm.

Data were represented as mean ± SD. ns, not significant; *, $p < 0.05$; **, $p < 0.01$; ***, $p < 0.001$.

Figure S7

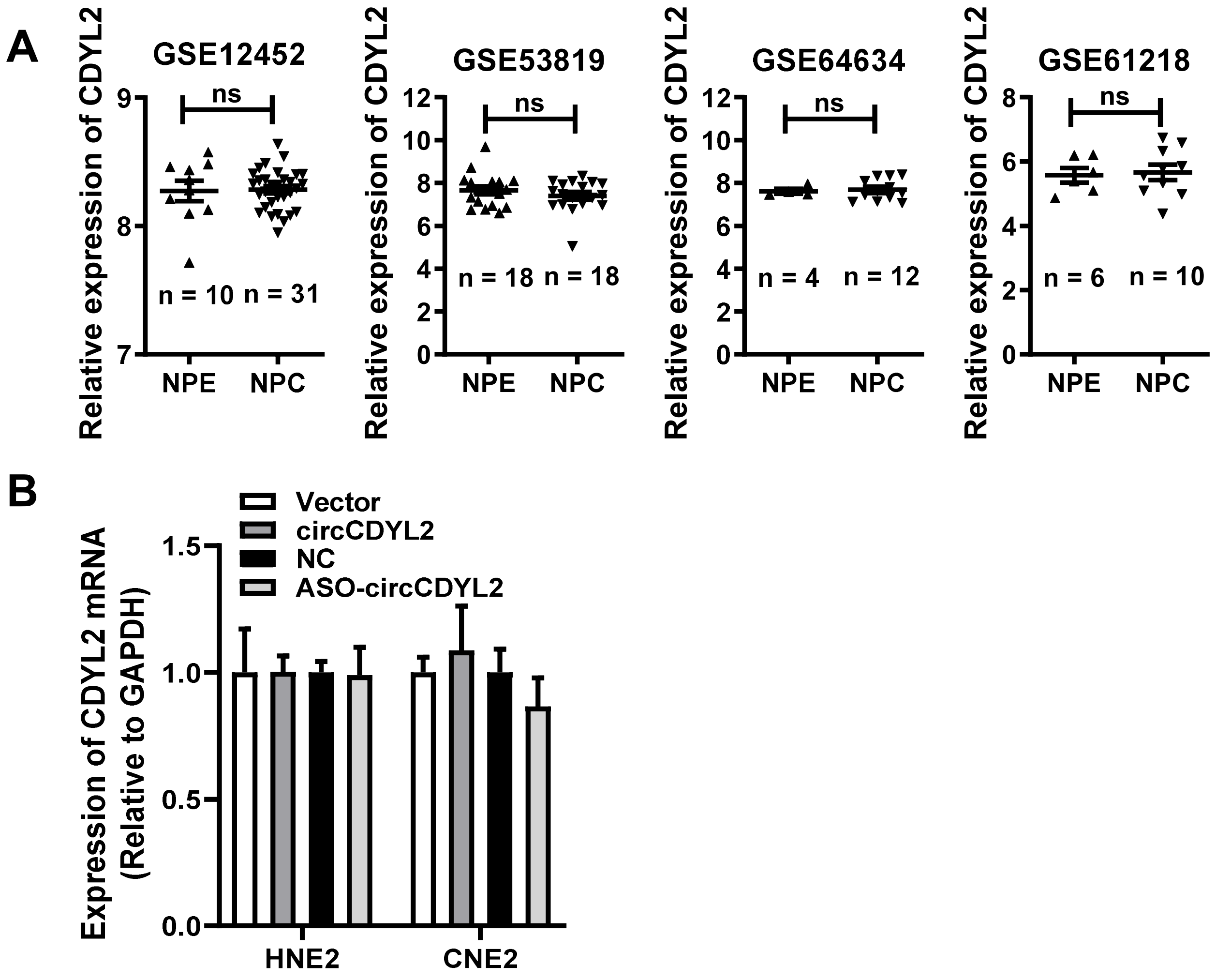

A
GSE12452
GSE53819
GSE64634
GSE61218
ns
Relative expression of CDYL2
n = 10
n = 31
n = 18
n = 18
n = 4
n = 12
n = 6
n = 10
NPE
NPC
B
Vector
circCDYL2
NC
ASO-circCDYL2
Expression of CDYL2 mRNA (Relative to GAPDH)
HNE2
CNE2

Supplemental Figure 7. circCDYL2 does not influence the expression of CDYL2.

A. The expression of CDYL2 was analyzed in NPC GEO datasets (GSE12452, GSE53819, GSE64634, and GSE61218).

B. The expression of CDYL2 mRNA was detected by RT-qPCR in HNE2 and CNE2 cells after overexpression or knockdown of circCDYL2.

Data were represented as mean ± SD. ns, not significant; *, $p < 0.05$; **, $p < 0.01$; ***, $p < 0.001$.

Figure S8

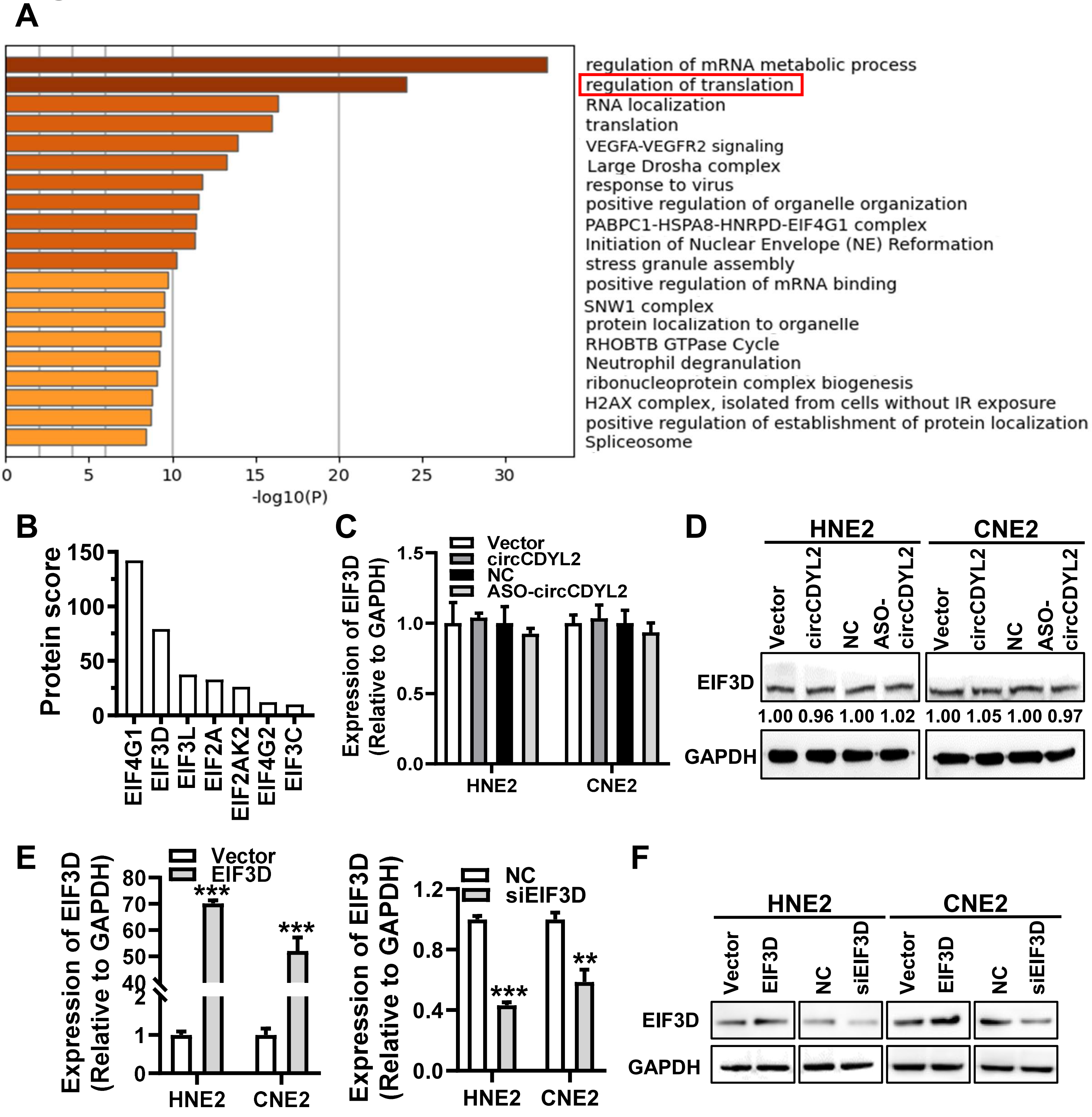

A
regulation of mRNA metabolic process
regulation of translation
RNA localization
translation
VEGFA-VEGFR2 signaling
Large Drosha complex
response to virus
positive regulation of organelle organization
PABPC1-HSPA8-HNRPD-EIF4G1 complex
Initiation of Nuclear Envelope (NE) Reformation
stress granule assembly
positive regulation of mRNA binding
SNW1 complex
protein localization to organelle
RHOBTB GTPase Cycle
Neutrophil degranulation
ribonucleoprotein complex biogenesis
H2AX complex, isolated from cells without IR exposure
positive regulation of establishment of protein localization
Spliceosome
-log10(P)
B
Protein score
EIF4G1
EIF3D
EIF3L
EIF2A
EIF2AK2
EIF4G2
EIF3C
C
Expression of EIF3D (Relative to GAPDH)
Vector
circCDYL2
NC
ASO-circCDYL2
HNE2
CNE2
D
HNE2
CNE2
Vector
circCDYL2
NC
ASO-circCDYL2
EIF3D
1.00 0.96 1.00 1.02
1.00 1.05 1.00 0.97
GAPDH
E
Expression of EIF3D (Relative to GAPDH)
Vector
EIF3D
***
***
HNE2
CNE2
NC
siEIF3D
***
**
F
HNE2
CNE2
Vector
EIF3D
NC
siEIF3D
EIF3D
GAPDH

Supplemental Figure 8. circCDYL2 interacts with EIF3D but does not regulate its expression.

A. The biotin-labeled circCDYL2 probe was used for RNA pulldown, and LC-MS/MS was used to identify proteins interacting with circCDYL2 in CNE2 cells. Pathways regulated by circCDYL2 were enriched using the GO database.

B. Translation initiation factors, including EIF4G1, EIF3D, EIF3L, EIF2A, EIF2AK2, EIF4G2, and EIF3C, were ranked based on their score values from mass spectrometry data.

C. RT-qPCR showed that circCDYL2 did not regulate the expression of EIF3D mRNA in HNE2 and CNE2 cells after overexpression or knockdown of circCDYL2.

D. Western blotting demonstrated that circCDYL2 did not regulate the expression of EIF3D protein in HNE2 and CNE2 cells after overexpression or knockdown of circCDYL2.

E. The transfection efficiency of EIF3D was detected by RT-qPCR in NPC cells after overexpression or knockdown of EIF3D.

F. The transfection efficiency of EIF3D was detected by western blotting in NPC cells after overexpression or knockdown of EIF3D.

Data were represented as mean ± SD. ns, not significant; *, $p < 0.05$; **, $p < 0.01$; ***, $p < 0.001$.

Figure S9

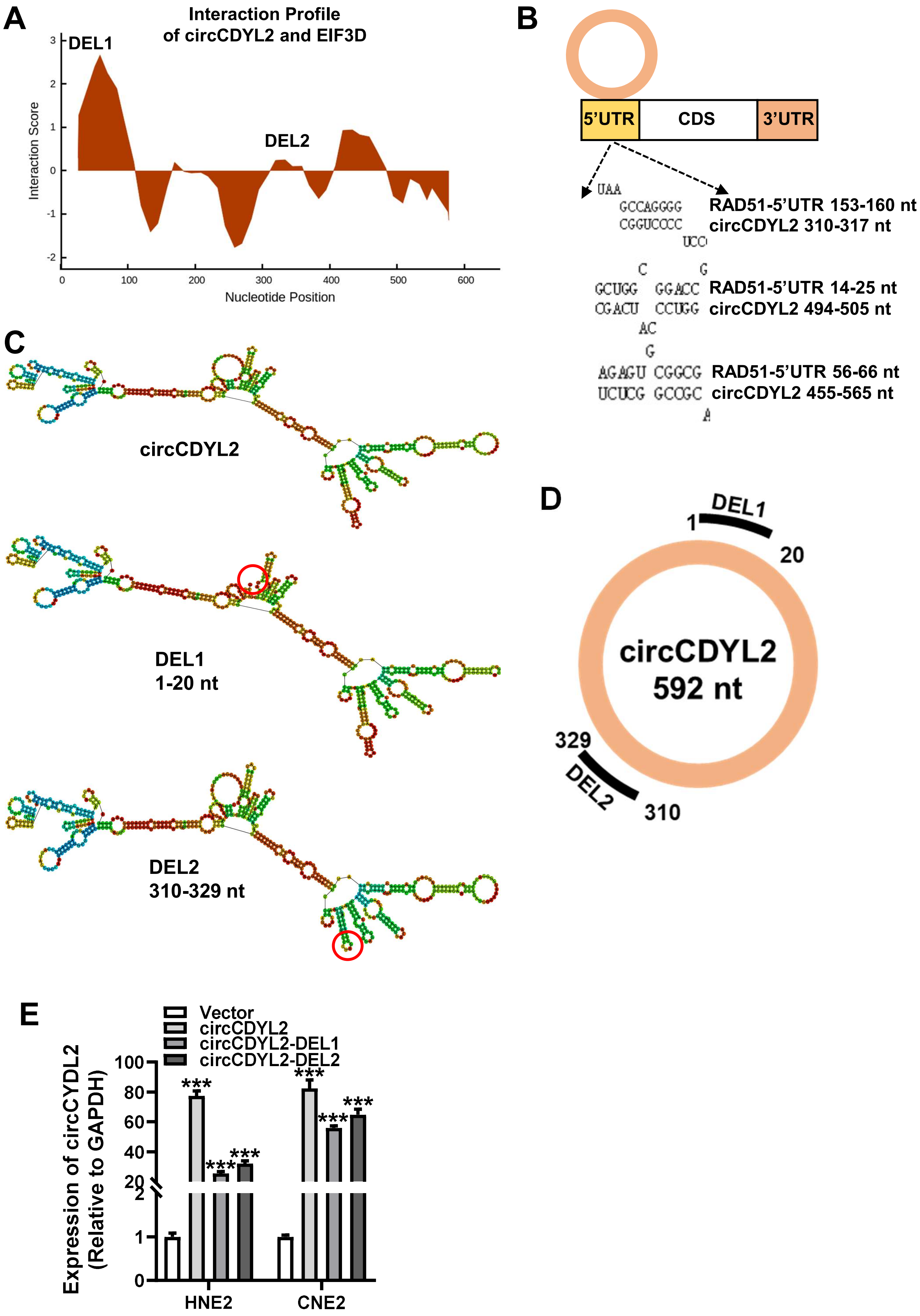
A
Interaction Profile
of circCDYL2 and EIF3D
DEL1
DEL2
Interaction Score
Nucleotide Position
B
5'UTR
CDS
3'UTR
RAD51-5'UTR 153-160 nt
circCDYL2 310-317 nt
RAD51-5'UTR 14-25 nt
circCDYL2 494-505 nt
RAD51-5'UTR 56-66 nt
circCDYL2 455-565 nt
C
circCDYL2
DEL1
1-20 nt
DEL2
310-329 nt
D
DEL1
1
20
circCDYL2
592 nt
329
DEL2
310
E
Vector
circCDYL2
circCDYL2-DEL1
circCDYL2-DEL2
Expression of circCYDL2
(Relative to GAPDH)
HNE2
CNE2

Supplemental Figure 9. circCDYL2 interacts with both EIF3D protein and RAD51 mRNA.

A. The binding site of circCDYL2 to EIF3D protein was predicted by the catRAPID software.

B. The binding site of circCDYL2 to RAD51 mRNA was predicted by the RNA hybrid software.

C. Two mutants of circCDYL2 (DEL1 and DEL2) were designed according to the secondary structure of circCDYL2 indicated by the RNA fold software.

D. Schematic diagram of the deleted regions of the circCDYL2 mutants.

E. The transfection efficiency of circCDYL2 was examined by qRT-PCR in HNE2 and CNE2 cells after overexpression of circCDYL2 or its deletion mutants (DEL1 and DEL2).

Data were represented as mean ± SD. ns, not significant; *, $p < 0.05$; **, $p < 0.01$; ***, $p < 0.001$.

Supplemental Table 1. Clinicopathological data of 45 NPC and 23 NPE tissues measured by RT-qPCR.

Patient No.	Gender (M=Male F=Female)	WHO histological diagnosis	T stage	N stage	M stage	Clinical Stages
Pat 01	M	non-tumor nasopharyngeal epithelial tissues	NA	NA	NA	NA
Pat 02	M	non-tumor nasopharyngeal epithelial tissues	NA	NA	NA	NA
Pat 03	M	non-tumor nasopharyngeal epithelial tissues	NA	NA	NA	NA
Pat 04	M	non-tumor nasopharyngeal epithelial tissues	NA	NA	NA	NA
Pat 05	F	non-tumor nasopharyngeal epithelial tissues	NA	NA	NA	NA
Pat 06	M	non-tumor nasopharyngeal epithelial tissues	NA	NA	NA	NA
Pat 07	M	non-tumor nasopharyngeal epithelial tissues	NA	NA	NA	NA
Pat 08	F	non-tumor nasopharyngeal epithelial tissues	NA	NA	NA	NA
Pat 09	M	non-tumor nasopharyngeal epithelial tissues	NA	NA	NA	NA
Pat 10	M	non-tumor nasopharyngeal epithelial tissues	NA	NA	NA	NA
Pat 11	F	non-tumor nasopharyngeal epithelial tissues	NA	NA	NA	NA
Pat 12	F	non-tumor nasopharyngeal epithelial tissues	NA	NA	NA	NA
Pat 13	M	non-tumor nasopharyngeal epithelial tissues	NA	NA	NA	NA
Pat 14	F	non-tumor nasopharyngeal epithelial tissues	NA	NA	NA	NA
Pat 15	M	non-tumor nasopharyngeal epithelial tissues	NA	NA	NA	NA
Pat 16	F	non-tumor nasopharyngeal epithelial tissues	NA	NA	NA	NA
Pat 17	M	non-tumor nasopharyngeal epithelial tissues	NA	NA	NA	NA
Pat 18	F	non-tumor nasopharyngeal epithelial tissues	NA	NA	NA	NA
Pat 19	M	non-tumor nasopharyngeal epithelial tissues	NA	NA	NA	NA
Pat 20	F	non-tumor nasopharyngeal epithelial tissues	NA	NA	NA	NA
Pat 21	M	non-tumor nasopharyngeal epithelial tissues	NA	NA	NA	NA
Pat 22	F	non-tumor nasopharyngeal epithelial tissues	NA	NA	NA	NA
Pat 23	M	non-tumor nasopharyngeal epithelial tissues	NA	NA	NA	NA
Pat 24	M	nasopharyngeal squamous cell carcinoma	2	0	0	II
Pat 25	M	nasopharyngeal squamous cell carcinoma	2	1	0	II
Pat 26	F	nasopharyngeal squamous cell carcinoma	2	1	0	II
Pat 27	M	nasopharyngeal squamous cell carcinoma	2	1	0	II
Pat 28	M	nasopharyngeal squamous cell carcinoma	3	2	0	III
Pat 29	M	nasopharyngeal squamous cell carcinoma	3	2	0	III
Pat 30	M	nasopharyngeal squamous cell carcinoma	1	2	0	III
Pat 31	M	nasopharyngeal squamous cell carcinoma	3	2	0	III
Pat 32	M	nasopharyngeal squamous cell carcinoma	2	2	0	III
Pat 33	F	nasopharyngeal squamous cell carcinoma	2	2	0	III
Pat 34	M	nasopharyngeal squamous cell carcinoma	3	1	0	III
Pat 35	M	nasopharyngeal squamous cell carcinoma	2	2	0	III
Pat 36	F	nasopharyngeal squamous cell carcinoma	2	2	0	III

Pat 37	F	nasopharyngeal squamous cell carcinoma	3	1	0	III
Pat 38	M	nasopharyngeal squamous cell carcinoma	3	2	0	III
Pat 39	M	nasopharyngeal squamous cell carcinoma	2	2	0	III
Pat 40	M	nasopharyngeal squamous cell carcinoma	3	2	0	III
Pat 41	F	nasopharyngeal squamous cell carcinoma	3	2	0	III
Pat 42	M	nasopharyngeal squamous cell carcinoma	3	3	0	IV
Pat 43	M	nasopharyngeal squamous cell carcinoma	3	3	0	IV
Pat 44	M	nasopharyngeal squamous cell carcinoma	4	2	0	IV
Pat 45	M	nasopharyngeal squamous cell carcinoma	2	3	0	IV
Pat 46	M	nasopharyngeal squamous cell carcinoma	2	3	0	IV
Pat 47	M	nasopharyngeal squamous cell carcinoma	2	3	0	IV
Pat 48	F	nasopharyngeal squamous cell carcinoma	4	3	0	IV
Pat 49	F	nasopharyngeal squamous cell carcinoma	3	3	0	IV
Pat 50	M	nasopharyngeal squamous cell carcinoma	4	3	0	IV
Pat 51	F	nasopharyngeal squamous cell carcinoma	3	3	0	IV
Pat 52	M	nasopharyngeal squamous cell carcinoma	2	3	0	IVa
Pat 53	M	nasopharyngeal squamous cell carcinoma	2	3	0	IVa
Pat 54	M	nasopharyngeal squamous cell carcinoma	4	2	0	IVa
Pat 55	F	nasopharyngeal squamous cell carcinoma	3	3	0	IVa
Pat 56	M	nasopharyngeal squamous cell carcinoma	2	3	0	IVa
Pat 57	M	nasopharyngeal squamous cell carcinoma	2	3	0	IVa
Pat 58	M	nasopharyngeal squamous cell carcinoma	2	3	0	IVa
Pat 59	M	nasopharyngeal squamous cell carcinoma	3	3	0	IVa
Pat 60	M	nasopharyngeal squamous cell carcinoma	3	3	0	IVa
Pat 61	F	nasopharyngeal squamous cell carcinoma	2	3	0	IVa
Pat 62	M	nasopharyngeal squamous cell carcinoma	4	2	0	IVa
Pat 63	M	nasopharyngeal squamous cell carcinoma	2	3	0	IVa
Pat 64	M	nasopharyngeal squamous cell carcinoma	2	3	0	IVa
Pat 65	M	nasopharyngeal squamous cell carcinoma	3	3	1	IVb
Pat 66	M	nasopharyngeal squamous cell carcinoma	3	3	0	IVb
Pat 67	F	nasopharyngeal squamous cell carcinoma	3	3	1	IVb
Pat 68	M	nasopharyngeal squamous cell carcinoma	3	2	1	IVb

Supplemental Table 2.Clinicopathological data of 203 paraffin-embedded NPC tissues and 56 non-neoplastic nasopharyngeal epithelial tissues for in situ hybridization.

Patient No.	Gender (M=Male F=Female)	Age at Diagnosis	Overall survival	WHO histological diagnosis	T stage	N stage	M stage	Clinical Stages	In situ hybridization score of circCDYL2		
									1	2	average
N1	M	45	36	non-tumor nasopharyngeal epithelial tissues	NA	NA	NA	NA	0	0	0
N2	M	44	21	non-tumor nasopharyngeal epithelial tissues	NA	NA	NA	NA	0	0	0
N3	M	19	23	non-tumor nasopharyngeal epithelial tissues	NA	NA	NA	NA	0	0	0
N4	M	59	83	non-tumor nasopharyngeal epithelial tissues	NA	NA	NA	NA	0	0	0
N5	M	51	111	non-tumor nasopharyngeal epithelial tissues	NA	NA	NA	NA	0	0	0
N6	M	40	30	non-tumor nasopharyngeal epithelial tissues	NA	NA	NA	NA	0	0	0
N7	F	38	78	non-tumor nasopharyngeal epithelial tissues	NA	NA	NA	NA	0	0	0
N8	M	39	78	non-tumor nasopharyngeal epithelial tissues	NA	NA	NA	NA	0	0	0
N9	M	32	73	non-tumor nasopharyngeal epithelial tissues	NA	NA	NA	NA	0	0	0
N10	M	64	72	non-tumor nasopharyngeal epithelial tissues	NA	NA	NA	NA	0	0	0
N11	M	42	71	non-tumor nasopharyngeal epithelial tissues	NA	NA	NA	NA	0	0	0
N12	M	24	70	non-tumor nasopharyngeal epithelial tissues	NA	NA	NA	NA	0	0	0
N13	F	39	65	non-tumor nasopharyngeal epithelial tissues	NA	NA	NA	NA	0	0	0
N14	F	58	6	non-tumor nasopharyngeal epithelial tissues	NA	NA	NA	NA	0	0	0
N15	M	48	115	non-tumor nasopharyngeal epithelial tissues	NA	NA	NA	NA	0	0	0
N16	M	57	9	non-tumor nasopharyngeal epithelial tissues	NA	NA	NA	NA	0	0	0
N17	M	75	14	non-tumor nasopharyngeal epithelial tissues	NA	NA	NA	NA	0	0	0
N18	M	47	16	non-tumor nasopharyngeal epithelial tissues	NA	NA	NA	NA	0	0	0
N19	M	50	31	non-tumor nasopharyngeal epithelial tissues	NA	NA	NA	NA	0	0	0
N20	M	22	35	non-tumor nasopharyngeal epithelial tissues	NA	NA	NA	NA	0	0	0
N21	M	43	36	non-tumor nasopharyngeal epithelial tissues	NA	NA	NA	NA	0	0	0
N22	F	63	45	non-tumor nasopharyngeal epithelial tissues	NA	NA	NA	NA	0	0	0
N23	M	31	48	non-tumor nasopharyngeal epithelial tissues	NA	NA	NA	NA	0	0	0
N24	F	50	62	non-tumor nasopharyngeal epithelial tissues	NA	NA	NA	NA	0	0	0
N25	M	52	65	non-tumor nasopharyngeal epithelial tissues	NA	NA	NA	NA	0	0	0
N26	M	63	74	non-tumor nasopharyngeal epithelial tissues	NA	NA	NA	NA	0	0	0
N27	M	43	76	non-tumor nasopharyngeal epithelial tissues	NA	NA	NA	NA	0	0	0
N28	M	47	105	non-tumor nasopharyngeal epithelial tissues	NA	NA	NA	NA	0	0	0
N29	M	43	109	non-tumor nasopharyngeal epithelial tissues	NA	NA	NA	NA	0	0	0
N30	M	37	84	non-tumor nasopharyngeal epithelial tissues	NA	NA	NA	NA	0	1	0.5
N31	M	30	38	non-tumor nasopharyngeal epithelial tissues	NA	NA	NA	NA	1	1	1

N32	M	44	23	non-tumor nasopharyngeal epithelial tissues	NA	NA	NA	NA	1	1	1
N33	M	51	9	non-tumor nasopharyngeal epithelial tissues	NA	NA	NA	NA	1	1	1
N34	F	34	48	non-tumor nasopharyngeal epithelial tissues	NA	NA	NA	NA	1	1	1
N35	M	48	9	non-tumor nasopharyngeal epithelial tissues	NA	NA	NA	NA	1	1	1
N36	M	52	79	non-tumor nasopharyngeal epithelial tissues	NA	NA	NA	NA	1	1	1
N37	M	52	64	non-tumor nasopharyngeal epithelial tissues	NA	NA	NA	NA	1	1	1
N38	M	51	33	non-tumor nasopharyngeal epithelial tissues	NA	NA	NA	NA	1	1	1
N39	M	56	42	non-tumor nasopharyngeal epithelial tissues	NA	NA	NA	NA	1	1	1
N40	M	52	17	non-tumor nasopharyngeal epithelial tissues	NA	NA	NA	NA	1	1	1
N41	M	39	17	non-tumor nasopharyngeal epithelial tissues	NA	NA	NA	NA	1	1	1
N42	M	43	59	non-tumor nasopharyngeal epithelial tissues	NA	NA	NA	NA	1	1	1
N43	M	50	66	non-tumor nasopharyngeal epithelial tissues	NA	NA	NA	NA	1	1	1
N44	M	51	66	non-tumor nasopharyngeal epithelial tissues	NA	NA	NA	NA	1	1	1
N45	M	66	73	non-tumor nasopharyngeal epithelial tissues	NA	NA	NA	NA	1	1	1
N46	M	43	87	non-tumor nasopharyngeal epithelial tissues	NA	NA	NA	NA	1	1	1
N47	M	41	33	non-tumor nasopharyngeal epithelial tissues	NA	NA	NA	NA	1	2	1.5
N48	M	50	66	non-tumor nasopharyngeal epithelial tissues	NA	NA	NA	NA	1	2	1.5
N49	M	44	80	non-tumor nasopharyngeal epithelial tissues	NA	NA	NA	NA	1	2	1.5
N50	M	39	37	non-tumor nasopharyngeal epithelial tissues	NA	NA	NA	NA	2	2	2
N51	M	44	27	non-tumor nasopharyngeal epithelial tissues	NA	NA	NA	NA	2	2	2
N52	M	39	37	non-tumor nasopharyngeal epithelial tissues	NA	NA	NA	NA	2	2	2
N53	M	62	12	non-tumor nasopharyngeal epithelial tissues	NA	NA	NA	NA	2	2	2
N54	M	32	39	non-tumor nasopharyngeal epithelial tissues	NA	NA	NA	NA	2	2	2
N55	M	38	73	non-tumor nasopharyngeal epithelial tissues	NA	NA	NA	NA	2	2	2
N56	F	50	93	non-tumor nasopharyngeal epithelial tissues	NA	NA	NA	NA	2	2	2
T1	1	62	118	nasopharyngeal squamous cell carcinoma	**1**	**0**	**0**	**I**	0	0	0
T2	1	48	115	nasopharyngeal squamous cell carcinoma	**2**	**2**	**0**	**III**	0	0	0
T3	2	56	45	nasopharyngeal squamous cell carcinoma	**2**	**1**	**0**	**II**	0	0	0
T4	1	50	99	nasopharyngeal squamous cell carcinoma	**2**	**1**	**1**	**IVb**	0	0	0
T5	1	49	108	nasopharyngeal squamous cell carcinoma	**2**	**1**	**0**	**II**	0	0	0
T6	1	37	114	nasopharyngeal squamous cell carcinoma	**2**	**1**	**0**	**II**	0	0	0
T7	2	42	117	nasopharyngeal squamous cell carcinoma	**2**	**1**	**0**	**II**	0	0	0
T8	1	24	118	nasopharyngeal squamous cell carcinoma	**2**	**0**	**0**	**II**	0	0	0
T9	1	46	69	nasopharyngeal squamous cell carcinoma	**3**	**2**	**1**	**IVb**	0	0	0
T10	1	33	88	nasopharyngeal squamous cell carcinoma	**3**	**2**	**0**	**III**	0	0	0
T11	2	53	12	nasopharyngeal squamous cell carcinoma	**3**	**0**	**1**	**IVb**	0	0	0
T12	1	43	36	nasopharyngeal squamous cell carcinoma	**2**	**2**	**1**	**IVb**	0	0	0
T13	1	44	21	nasopharyngeal squamous cell carcinoma	**2**	**0**	**0**	**II**	0	0	0

T14	1	57	24	nasopharyngeal squamous cell carcinoma	**3**	**2**	**1**	**IVb**	0	0	0
T15	1	52	79	nasopharyngeal squamous cell carcinoma	**1**	**2**	**0**	**III**	0	0	0
T16	1	49	74	nasopharyngeal squamous cell carcinoma	**3**	**0**	**0**	**III**	0	0	0
T17	1	70	66	nasopharyngeal squamous cell carcinoma	**2**	**2**	**0**	**III**	0	0	0
T18	2	49	111	nasopharyngeal squamous cell carcinoma	**2**	**0**	**0**	**II**	0	1	0.5
T19	1	37	123	nasopharyngeal squamous cell carcinoma	**2**	**1**	**0**	**II**	0	1	0.5
T20	1	56	42	nasopharyngeal squamous cell carcinoma	**1**	**2**	**0**	**III**	1	1	1
T21	2	32	123	nasopharyngeal squamous cell carcinoma	**1**	**1**	**0**	**II**	1	1	1
T22	2	51	12	nasopharyngeal squamous cell carcinoma	**2**	**2**	**0**	**III**	1	1	1
T23	1	53	41	nasopharyngeal squamous cell carcinoma	**2**	**1**	**0**	**II**	1	1	1
T24	1	55	68	nasopharyngeal squamous cell carcinoma	**2**	**2**	**0**	**III**	1	1	1
T25	1	50	104	nasopharyngeal squamous cell carcinoma	**2**	**0**	**0**	**II**	1	1	1
T26	1	31	112	nasopharyngeal squamous cell carcinoma	**2**	**3**	**1**	**IVb**	1	1	1
T27	1	31	114	nasopharyngeal squamous cell carcinoma	**2**	**0**	**0**	**II**	1	1	1
T28	1	56	117	nasopharyngeal squamous cell carcinoma	**2**	**1**	**0**	**II**	1	1	1
T29	2	48	73	nasopharyngeal squamous cell carcinoma	**3**	**2**	**0**	**III**	1	1	1
T30	1	47	76	nasopharyngeal squamous cell carcinoma	**3**	**0**	**0**	**III**	1	1	1
T31	1	67	98	nasopharyngeal squamous cell carcinoma	**3**	**1**	**0**	**III**	1	1	1
T32	1	48	118	nasopharyngeal squamous cell carcinoma	**3**	**1**	**0**	**III**	1	1	1
T33	1	39	37	nasopharyngeal squamous cell carcinoma	**2**	**0**	**0**	**II**	1	1	1
T34	1	50	36	nasopharyngeal squamous cell carcinoma	**4**	**2**	**0**	**IVa**	1	1	1
T35	1	45	36	nasopharyngeal squamous cell carcinoma	**3**	**0**	**0**	**III**	1	1	1
T36	1	37	35	nasopharyngeal squamous cell carcinoma	**2**	**3**	**0**	**IVa**	1	1	1
T37	1	32	14	nasopharyngeal squamous cell carcinoma	**3**	**3**	**0**	**IVa**	1	1	1
T38	1	41	33	nasopharyngeal squamous cell carcinoma	**4**	**2**	**0**	**IVa**	1	1	1
T39	1	44	23	nasopharyngeal squamous cell carcinoma	**4**	**2**	**0**	**IVa**	1	1	1
T40	1	35	33	nasopharyngeal squamous cell carcinoma	**2**	**2**	**0**	**III**	1	1	1
T41	2	45	32	nasopharyngeal squamous cell carcinoma	**3**	**0**	**1**	**IVb**	1	1	1
T42	1	19	23	nasopharyngeal squamous cell carcinoma	**4**	**2**	**1**	**IVb**	1	1	1
T43	1	47	6	nasopharyngeal squamous cell carcinoma	**4**	**3**	**0**	**IVa**	1	1	1
T44	1	51	9	nasopharyngeal squamous cell carcinoma	**2**	**3**	**0**	**IVa**	1	1	1
T45	1	39	37	nasopharyngeal squamous cell carcinoma	**4**	**1**	**1**	**IVb**	1	1	1
T46	1	39	37	nasopharyngeal squamous cell carcinoma	**4**	**1**	**0**	**IVa**	1	1	1
T47	2	62	30	nasopharyngeal squamous cell carcinoma	**2**	**2**	**0**	**III**	1	1	1
T48	1	59	83	nasopharyngeal squamous cell carcinoma	**3**	**1**	**0**	**III**	1	1	1
T49	1	51	111	nasopharyngeal squamous cell carcinoma	**2**	**1**	**0**	**II**	1	1	1
T50	1	39	78	nasopharyngeal squamous cell carcinoma	**2**	**2**	**0**	**III**	1	1	1
T51	1	68	77	nasopharyngeal squamous cell carcinoma	**1**	**1**	**0**	**II**	1	1	1

T52	1	38	73	nasopharyngeal squamous cell carcinoma	**2**	**2**	**0**	**III**	1	1	1
T53	1	64	72	nasopharyngeal squamous cell carcinoma	**2**	**0**	**0**	**II**	1	1	1
T54	1	42	71	nasopharyngeal squamous cell carcinoma	**2**	**2**	**0**	**III**	1	1	1
T55	1	39	38	nasopharyngeal squamous cell carcinoma	**1**	**2**	**0**	**III**	1	1	1
T56	2	39	65	nasopharyngeal squamous cell carcinoma	**2**	**0**	**0**	**II**	1	1	1
T57	1	43	64	nasopharyngeal squamous cell carcinoma	**1**	**0**	**0**	**I**	1	1	1
T58	1	71	52	nasopharyngeal squamous cell carcinoma	**1**	**0**	**0**	**I**	1	2	1.5
T59	1	47	104	nasopharyngeal squamous cell carcinoma	**1**	**0**	**0**	**I**	1	2	1.5
T60	1	73	111	nasopharyngeal squamous cell carcinoma	**1**	**1**	**0**	**II**	1	2	1.5
T61	1	36	13	nasopharyngeal squamous cell carcinoma	**2**	**2**	**0**	**III**	1	2	1.5
T62	1	51	34	nasopharyngeal squamous cell carcinoma	**2**	**2**	**0**	**III**	1	2	1.5
T63	2	58	6	nasopharyngeal squamous cell carcinoma	**3**	**1**	**0**	**III**	1	2	1.5
T64	1	51	33	nasopharyngeal squamous cell carcinoma	**3**	**3**	**0**	**IVa**	1	2	1.5
T65	1	40	20	nasopharyngeal squamous cell carcinoma	**2**	**0**	**0**	**II**	2	1	1.5
T66	1	47	34	nasopharyngeal squamous cell carcinoma	**3**	**3**	**0**	**IVa**	1	2	1.5
T67	1	45	6	nasopharyngeal squamous cell carcinoma	**3**	**2**	**0**	**III**	2	1	1.5
T68	2	49	108	nasopharyngeal squamous cell carcinoma	**1**	**0**	**0**	**I**	2	2	2
T69	1	47	105	nasopharyngeal squamous cell carcinoma	**2**	**3**	**1**	**IVb**	2	2	2
T70	1	31	48	nasopharyngeal squamous cell carcinoma	**2**	**2**	**0**	**III**	2	2	2
T71	1	66	5	nasopharyngeal squamous cell carcinoma	**2**	**3**	**0**	**IVa**	2	2	2
T72	1	63	5	nasopharyngeal squamous cell carcinoma	**2**	**2**	**1**	**IVb**	2	2	2
T73	1	51	5	nasopharyngeal squamous cell carcinoma	**2**	**2**	**1**	**IVb**	2	2	2
T74	2	46	10	nasopharyngeal squamous cell carcinoma	**2**	**2**	**0**	**III**	2	2	2
T75	1	45	14	nasopharyngeal squamous cell carcinoma	**2**	**3**	**0**	**IVa**	2	2	2
T76	1	62	91	nasopharyngeal squamous cell carcinoma	**2**	**1**	**0**	**II**	2	2	2
T77	1	58	101	nasopharyngeal squamous cell carcinoma	**2**	**0**	**0**	**II**	2	2	2
T78	2	52	71	nasopharyngeal squamous cell carcinoma	**2**	**2**	**0**	**III**	2	2	2
T79	1	43	109	nasopharyngeal squamous cell carcinoma	**3**	**3**	**0**	**IVa**	2	2	2
T80	1	37	84	nasopharyngeal squamous cell carcinoma	**3**	**2**	**0**	**III**	2	2	2
T81	1	43	76	nasopharyngeal squamous cell carcinoma	**3**	**0**	**0**	**III**	2	2	2
T82	1	22	35	nasopharyngeal squamous cell carcinoma	**3**	**1**	**0**	**III**	2	2	2
T83	1	50	31	nasopharyngeal squamous cell carcinoma	**3**	**1**	**0**	**III**	2	2	2
T84	1	66	73	nasopharyngeal squamous cell carcinoma	**3**	**3**	**0**	**IVa**	2	2	2
T85	1	50	66	nasopharyngeal squamous cell carcinoma	**3**	**1**	**1**	**IVb**	2	2	2
T86	2	65	49	nasopharyngeal squamous cell carcinoma	**3**	**2**	**0**	**III**	2	2	2
T87	2	45	62	nasopharyngeal squamous cell carcinoma	**3**	**3**	**0**	**IVa**	2	2	2
T88	1	56	77	nasopharyngeal squamous cell carcinoma	**3**	**3**	**0**	**IVa**	2	2	2
T89	1	75	14	nasopharyngeal squamous cell carcinoma	**4**	**2**	**0**	**IVa**	2	2	2

T90	1	44	80	nasopharyngeal squamous cell carcinoma	**4**	**1**	**0**	**IVa**	2	2	2
T91	2	52	32	nasopharyngeal squamous cell carcinoma	**4**	**1**	**1**	**IVb**	2	2	2
T92	1	30	38	nasopharyngeal squamous cell carcinoma	**2**	**2**	**0**	**III**	2	2	2
T93	1	41	8	nasopharyngeal squamous cell carcinoma	**2**	**2**	**0**	**III**	2	2	2
T94	2	60	37	nasopharyngeal squamous cell carcinoma	**4**	**2**	**1**	**IVb**	2	2	2
T95	2	52	37	nasopharyngeal squamous cell carcinoma	**3**	**1**	**0**	**III**	2	2	2
T96	1	67	35	nasopharyngeal squamous cell carcinoma	**4**	**3**	**0**	**IVa**	2	2	2
T97	1	49	34	nasopharyngeal squamous cell carcinoma	**2**	**3**	**0**	**IVa**	2	2	2
T98	2	37	34	nasopharyngeal squamous cell carcinoma	**3**	**2**	**0**	**III**	2	2	2
T99	1	52	32	nasopharyngeal squamous cell carcinoma	**3**	**2**	**0**	**III**	2	2	2
T100	1	44	27	nasopharyngeal squamous cell carcinoma	**2**	**2**	**0**	**III**	2	2	2
T101	1	44	27	nasopharyngeal squamous cell carcinoma	**2**	**2**	**0**	**III**	2	2	2
T102	2	47	36	nasopharyngeal squamous cell carcinoma	**1**	**3**	**0**	**IVa**	2	2	2
T103	1	54	4	nasopharyngeal squamous cell carcinoma	**2**	**2**	**0**	**III**	2	2	2
T104	2	48	41	nasopharyngeal squamous cell carcinoma	**2**	**2**	**0**	**III**	2	2	2
T105	1	62	12	nasopharyngeal squamous cell carcinoma	**1**	**3**	**1**	**IVb**	2	2	2
T106	1	58	21	nasopharyngeal squamous cell carcinoma	**2**	**2**	**0**	**III**	2	2	2
T107	2	38	78	nasopharyngeal squamous cell carcinoma	**2**	**2**	**0**	**III**	2	2	2
T108	1	54	73	nasopharyngeal squamous cell carcinoma	**2**	**1**	**0**	**II**	2	2	2
T109	2	42	73	nasopharyngeal squamous cell carcinoma	**2**	**1**	**0**	**II**	2	2	2
T110	1	24	70	nasopharyngeal squamous cell carcinoma	**4**	**0**	**0**	**IVa**	2	2	2
T111	2	38	43	nasopharyngeal squamous cell carcinoma	**2**	**1**	**0**	**II**	2	2	2
T112	1	59	38	nasopharyngeal squamous cell carcinoma	**2**	**2**	**0**	**III**	2	2	2
T113	1	39	56	nasopharyngeal squamous cell carcinoma	**2**	**2**	**0**	**III**	2	2	2
T114	1	43	36	nasopharyngeal squamous cell carcinoma	**1**	**3**	**1**	**IVb**	3	2	2.5
T115	2	50	93	nasopharyngeal squamous cell carcinoma	**1**	**0**	**0**	**I**	2	3	2.5
T116	2	51	93	nasopharyngeal squamous cell carcinoma	**1**	**0**	**0**	**I**	3	2	2.5
T117	1	27	98	nasopharyngeal squamous cell carcinoma	**1**	**0**	**0**	**I**	3	2	2.5
T118	1	43	87	nasopharyngeal squamous cell carcinoma	**1**	**0**	**0**	**I**	3	2	2.5
T119	1	52	17	nasopharyngeal squamous cell carcinoma	**2**	**2**	**0**	**III**	2	3	2.5
T120	1	27	9	nasopharyngeal squamous cell carcinoma	**2**	**3**	**0**	**IVa**	2	3	2.5
T121	1	44	44	nasopharyngeal squamous cell carcinoma	**2**	**2**	**0**	**III**	2	3	2.5
T122	1	45	38	nasopharyngeal squamous cell carcinoma	**2**	**2**	**0**	**III**	2	3	2.5
T123	1	52	57	nasopharyngeal squamous cell carcinoma	**2**	**2**	**0**	**III**	3	2	2.5
T124	2	50	62	nasopharyngeal squamous cell carcinoma	**3**	**2**	**0**	**III**	2	3	2.5
T125	1	49	30	nasopharyngeal squamous cell carcinoma	**3**	**1**	**1**	**IVb**	2	3	2.5
T126	1	39	85	nasopharyngeal squamous cell carcinoma	**3**	**2**	**0**	**III**	2	3	2.5
T127	1	32	106	nasopharyngeal squamous cell carcinoma	**3**	**2**	**0**	**III**	2	3	2.5

T128	1	64	48	nasopharyngeal squamous cell carcinoma	**4**	**0**	**0**	**IVa**	2	3	2.5
T129	1	56	33	nasopharyngeal squamous cell carcinoma	**3**	**2**	**0**	**III**	2	3	2.5
T130	2	36	102	nasopharyngeal squamous cell carcinoma	**2**	**2**	**0**	**III**	3	2	2.5
T131	1	50	66	nasopharyngeal squamous cell carcinoma	**3**	**1**	**0**	**III**	3	2	2.5
T132	1	57	9	nasopharyngeal squamous cell carcinoma	**1**	**3**	**1**	**IVb**	3	3	3
T133	1	62	43	nasopharyngeal squamous cell carcinoma	**1**	**3**	**1**	**IVb**	3	3	3
T134	1	60	70	nasopharyngeal squamous cell carcinoma	**1**	**0**	**0**	**I**	3	3	3
T135	1	37	47	nasopharyngeal squamous cell carcinoma	**1**	**1**	**0**	**II**	3	3	3
T136	2	63	45	nasopharyngeal squamous cell carcinoma	**2**	**2**	**0**	**III**	3	3	3
T137	1	43	59	nasopharyngeal squamous cell carcinoma	**2**	**2**	**0**	**III**	3	3	3
T138	1	39	17	nasopharyngeal squamous cell carcinoma	**2**	**2**	**0**	**III**	3	3	3
T139	2	51	3	nasopharyngeal squamous cell carcinoma	**2**	**3**	**0**	**IVa**	3	3	3
T140	1	54	13	nasopharyngeal squamous cell carcinoma	**2**	**3**	**1**	**IVb**	3	3	3
T141	1	39	16	nasopharyngeal squamous cell carcinoma	**2**	**2**	**1**	**IVb**	3	3	3
T142	1	39	19	nasopharyngeal squamous cell carcinoma	**2**	**3**	**1**	**IVb**	3	3	3
T143	1	46	31	nasopharyngeal squamous cell carcinoma	**2**	**2**	**1**	**IVb**	3	3	3
T144	2	52	43	nasopharyngeal squamous cell carcinoma	**2**	**2**	**0**	**III**	3	3	3
T145	1	65	47	nasopharyngeal squamous cell carcinoma	**2**	**2**	**0**	**III**	3	3	3
T146	1	46	66	nasopharyngeal squamous cell carcinoma	**2**	**3**	**0**	**IVa**	3	3	3
T147	1	69	102	nasopharyngeal squamous cell carcinoma	**2**	**0**	**0**	**II**	3	3	3
T148	2	51	109	nasopharyngeal squamous cell carcinoma	**2**	**1**	**0**	**II**	3	3	3
T149	1	41	114	nasopharyngeal squamous cell carcinoma	**2**	**0**	**0**	**II**	3	3	3
T150	1	62	123	nasopharyngeal squamous cell carcinoma	**2**	**0**	**0**	**II**	3	3	3
T151	1	49	21	nasopharyngeal squamous cell carcinoma	**2**	**3**	**0**	**III**	3	3	3
T152	1	63	74	nasopharyngeal squamous cell carcinoma	**3**	**2**	**0**	**III**	3	3	3
T153	1	52	65	nasopharyngeal squamous cell carcinoma	**3**	**0**	**0**	**III**	3	3	3
T154	1	51	66	nasopharyngeal squamous cell carcinoma	**3**	**0**	**0**	**III**	3	3	3
T155	1	66	1	nasopharyngeal squamous cell carcinoma	**3**	**3**	**1**	**IVb**	3	3	3
T156	1	33	10	nasopharyngeal squamous cell carcinoma	**3**	**2**	**0**	**III**	3	3	3
T157	1	52	61	nasopharyngeal squamous cell carcinoma	**3**	**0**	**0**	**III**	3	3	3
T158	1	34	68	nasopharyngeal squamous cell carcinoma	**3**	**0**	**0**	**III**	3	3	3
T159	2	47	89	nasopharyngeal squamous cell carcinoma	**3**	**2**	**0**	**III**	3	3	3
T160	1	47	16	nasopharyngeal squamous cell carcinoma	**4**	**0**	**0**	**IVa**	3	3	3
T161	1	38	13	nasopharyngeal squamous cell carcinoma	**4**	**2**	**1**	**IVb**	3	3	3
T162	1	76	21	nasopharyngeal squamous cell carcinoma	**4**	**0**	**0**	**IVa**	3	3	3
T163	2	49	38	nasopharyngeal squamous cell carcinoma	**2**	**2**	**1**	**IVb**	3	3	3
T164	2	39	9	nasopharyngeal squamous cell carcinoma	**4**	**2**	**1**	**IVb**	3	3	3
T165	1	38	34	nasopharyngeal squamous cell carcinoma	**2**	**1**	**1**	**IVb**	3	3	3

T166	1	46	33	nasopharyngeal squamous cell carcinoma	**3**	**1**	**1**	**IVb**	3	3	3
T167	2	46	33	nasopharyngeal squamous cell carcinoma	**4**	**1**	**0**	**IVa**	3	3	3
T168	1	42	28	nasopharyngeal squamous cell carcinoma	**4**	**2**	**0**	**IVa**	3	3	3
T169	1	56	5	nasopharyngeal squamous cell carcinoma	**4**	**2**	**0**	**IVa**	3	3	3
T170	1	38	10	nasopharyngeal squamous cell carcinoma	**3**	**0**	**0**	**III**	3	3	3
T171	1	55	72	nasopharyngeal squamous cell carcinoma	**1**	**0**	**0**	**I**	3	3	3
T172	1	39	71	nasopharyngeal squamous cell carcinoma	**2**	**3**	**0**	**IVa**	3	3	3
T173	1	49	75	nasopharyngeal squamous cell carcinoma	**3**	**2**	**0**	**III**	3	3	3
T174	2	34	48	nasopharyngeal squamous cell carcinoma	**3**	**1**	**0**	**III**	3	3	3
T175	2	42	102	nasopharyngeal squamous cell carcinoma	**4**	**2**	**0**	**IVa**	3	3	3
T176	1	40	30	nasopharyngeal squamous cell carcinoma	**3**	**1**	**0**	**III**	3	3	3
T177	1	45	68	nasopharyngeal squamous cell carcinoma	**3**	**1**	**0**	**III**	3	3	3
T178	1	48	35	nasopharyngeal squamous cell carcinoma	**3**	**3**	**0**	**IVa**	3	3	3
T179	1	37	55	nasopharyngeal squamous cell carcinoma	**2**	**2**	**0**	**III**	3	3	3
T180	2	56	38	nasopharyngeal squamous cell carcinoma	**3**	**1**	**0**	**III**	3	3	3
T181	1	37	77	nasopharyngeal squamous cell carcinoma	**2**	**2**	**0**	**III**	3	3	3
T182	1	48	9	nasopharyngeal squamous cell carcinoma	**2**	**3**	**0**	**IVa**	3	3	3
T183	1	60	21	nasopharyngeal squamous cell carcinoma	**1**	**1**	**0**	**II**	3	3	3
T184	1	27	10	nasopharyngeal squamous cell carcinoma	**3**	**2**	**0**	**III**	3	3	3
T185	1	32	39	nasopharyngeal squamous cell carcinoma	**2**	**2**	**0**	**III**	3	3	3
T186	1	39	87	nasopharyngeal squamous cell carcinoma	**2**	**2**	**0**	**III**	3	3	3
T187	1	66	80	nasopharyngeal squamous cell carcinoma	**2**	**2**	**0**	**III**	3	3	3
T188	1	61	81	nasopharyngeal squamous cell carcinoma	**3**	**2**	**0**	**III**	3	3	3
T189	1	46	81	nasopharyngeal squamous cell carcinoma	**2**	**2**	**0**	**III**	3	3	3
T190	1	58	71	nasopharyngeal squamous cell carcinoma	**3**	**0**	**0**	**III**	3	3	3
T191	1	47	75	nasopharyngeal squamous cell carcinoma	**4**	**2**	**0**	**IVa**	3	3	3
T192	1	54	74	nasopharyngeal squamous cell carcinoma	**2**	**1**	**1**	**IVb**	3	3	3
T193	1	35	20	nasopharyngeal squamous cell carcinoma	**2**	**2**	**0**	**III**	3	3	3
T194	1	32	73	nasopharyngeal squamous cell carcinoma	**2**	**3**	**0**	**IVa**	3	3	3
T195	2	50	72	nasopharyngeal squamous cell carcinoma	**3**	**2**	**0**	**III**	3	3	3
T196	1	59	60	nasopharyngeal squamous cell carcinoma	**2**	**1**	**0**	**II**	3	3	3
T197	1	42	69	nasopharyngeal squamous cell carcinoma	**1**	**0**	**0**	**I**	3	3	3
T198	1	53	69	nasopharyngeal squamous cell carcinoma	**2**	**2**	**0**	**III**	3	3	3
T199	1	60	17	nasopharyngeal squamous cell carcinoma	**2**	**2**	**0**	**III**	3	3	3
T200	1	23	37	nasopharyngeal squamous cell carcinoma	**3**	**2**	**0**	**III**	3	3	3
T201	1	52	64	nasopharyngeal squamous cell carcinoma	**2**	**1**	**0**	**II**	3	3	3
T202	1	49	62	nasopharyngeal squamous cell carcinoma	**3**	**0**	**0**	**III**	3	3	3
T203	1	45	31	nasopharyngeal squamous cell carcinoma	**1**	**0**	**0**	**I**	3	3	3

Supplemental Table 3. Primers, probes, and siRNA used in this study.

Primer names	sequence (5'-3')
circCDYL2-F	CCTGGCTTGGATTTGAATGA
circCDYL2-R	CTCCCGTAGCCTTTCCATC
CDYL2 mRNA-F	GCCAATGAGATGCTGTTCTGT
CDYL2 mRNA -R	CTCCTCTAACACCACGGCA
18S-F	TCTTAGCTGAGTGTCCCGCG
18S-R	ATCATGGCCTCAGTTCCGAA
GAPDH-F	CTGACTTCAACAGCGACACC
GAPDH-R	GTGGTCCAGGGGTCTTACTC
U6-F	CTCGCTTCGGCAGCACA
U6-R	AACGCTTCACGAATTTGCGT
BRCA1-F	AGCACTCTAGGGAAGGCAAA
BRCA1-R	ATGCTTGTTTCCCGACTGTG
53BP1-F	TGTGGTTCCATCAGTCAGGT
53BP1 -R	AACCCCAAAACCCAACTGTG
RAD51-F	GGAACTGCAACTCATCTGGG
RAD51-R	CATTGCCATTACTCGGTCCG
EIF3D-F	AGATCCTGCCTAAGAGTGCC
EIF3D-R	CACTCAATGTCCTGTGGCTC
circCDYL2-DEL1-F	GTGAATCACGCTACACTGGC
circCDYL2-DEL1-R	TGCTTCCCTGACTTGATCCT

F: Forword R: Reverse

Probes names	sequence (5'-3')
Dig-circCDYL2	Dig-TACAATCCTTTCAACCGAGCCCGTTCTCCG-Dig
Biotin-circCDYL2	Biotin-TACAATCCTTTCAACCGAGCCCGTTCTCCG-Biotin
Unbiotin-circCDYL2	TACAATCCTTTCAACCGAGCCCGTTCTCCG
Biotin-circCDYL2-DEL'	Biotin-TTCCATCGGATAAGACGAGCCCGTTCTCCG-Biotin

siRNA	Target sequences (5'-3')
ASO-circCDYL2	GAGAACGGGCTCGGTTGAAA
BRCA1 siRNA	GATAGTTCTACCAGTAAA
53BP1 siRNA	GCCAGGTTCTAGAGGATGA
RAD51-si1	GAAAGGCCATGTACATTGA
RAD51-si2	GACTGGATCTATCACAGAA
EIF3D siRNA	GGATCAGAAATCACAGAAA

Supplemental Table 4. Antibodies used in this study.

Antibodies	Application, dilution	Catalogue No.	Supplier
Phospho-Histone H2AX(γH2AX	WB,1:1000; IF,1:200	9718T	Cell Signaling Technology
H2AX	WB,1:1000;	10856-1-AP	Proteintech
RAD51	WB,1:1000; IHC,1:200	ab133534	Abcam
RAD51	IF,1:100	ab88572	Abcam
BRCA1	WB,1:1000;IF,1:200	9010T	Cell Signaling Technology
RPA1	WB,1:1000;IF,1:200	67973-1-Ig	Proteintech
Ku70	WB,1:1000;	ab92450	Abcam
NBS1	WB,1:1000;	81234T	Cell Signaling Technology
EIF3D	WB,1:1000; IF,1:100	10219-1-AP	Proteintech
EIF3L	WB,1:1000;	CSB-PA002298	CUSABIO
EIF4G1	WB,1:1000;	CSB-PA243767	CUSABIO
GAPDH	WB,1:5000;	60004-1-Ig	Proteintech

Supplemental Table 5. Potential circCDYL2 interacting proteins in HNE2 cells using the LC-MS/MS method after pulldown by the biotin-labeled circCDYL2 probe.

Accession	GENE	Score	Coverage	Area	MW [kDa]
M0QZM1	HNRNPM	456.75	73.11	1.06E+08	40.02
Q13283	G3BP1	418.69	54.08	6.96E+07	52.13
Q92499	DDX1	394.16	47.97	6.95E+07	82.38
P11940	PABPC1	383.62	38.21	1.16E+08	70.63
A0A2R8Y5	DDX3X	372.42	44.69	4.48E+07	69.00
P42166	TMPO	371.44	19.16	2.46E+08	75.45
P14618	PKM	338.87	45.57	1.02E+08	57.90
P02545	LMNA	336.55	37.50	3.64E+07	74.09
A6NLN1	PTBP1	285.26	36.43	1.21E+08	56.48
B3GQS7	HSPD1	272.04	34.80	3.72E+07	60.64
P11142	HSPA8	260.04	32.20	3.30E+07	70.85
A0A2R8Y6	ENO1	257.38	29.03	3.63E+07	47.30
Q7KYM9	ORF	253.96	38.95	2.25E+07	59.93
Q7Z417	NUFIP2	244.79	36.83	7.00E+07	76.07
M0QXS5	HNRNPL	226.60	35.28	2.18E+07	58.44
P07900	HSP90AA1	221.77	26.78	5.14E+07	84.61
Q9Y3I0	RTCB	218.15	35.84	3.58E+07	55.17
A0A024RD	G3BP2	212.64	31.63	2.40E+07	50.79
I6L9E8	FAM98A	201.30	27.03	2.82E+07	55.14
A0A1U9X7	HSPA1A	201.28	19.03	2.55E+07	70.00
K7ENG2	U2AF2	174.47	34.85	3.75E+07	33.88
A0A1X7SE	DDX17	169.02	17.83	9.00E+07	80.20
P50402	EMD	168.88	25.20	2.16E+07	28.98
B0QYK0	EWSR1	168.20	15.05	1.13E+08	64.89
P11021	HSPA5	155.59	31.50	2.07E+07	72.29
Q14568	HSP90AA2P	149.20	9.04	1.26E+06	39.34
A0A0F7KY	FXR1	147.08	32.50	1.96E+07	76.15
Q14694	USP10	144.97	22.18	3.18E+07	87.08
B5BUE6	DDX5	143.46	18.89	2.46E+07	69.08
E7EX73	EIF4G1	142.40	12.95	1.74E+07	158.55
J3KPE3	RACK1	137.97	47.99	2.17E+07	30.09
Q9NZI8	IGF2BP1	135.68	27.90	1.02E+07	63.44
B4DUR8	CCT3	130.37	23.20	6.83E+06	55.64
Q96I24	FUBP3	128.39	27.62	1.17E+07	61.60
Q05CK9	SYNCRIP	128.02	31.35	1.32E+07	50.62
H0Y6E7	RBMX	122.47	23.29	2.41E+07	31.84
Q04837	SSBP1	114.70	55.41	6.28E+07	17.25
A1JUI8	CCT6A	113.64	15.98	7.36E+06	53.65
Q9UG63	ABCF2	102.26	20.22	1.09E+07	71.24
P17987	TCP1	101.75	20.68	4.69E+06	60.31
O00425	IGF2BP3	98.50	19.69	5.21E+06	63.67
Q7Z759	CCT8	93.17	17.30	4.75E+06	54.07
P07355	ANXA2	83.98	21.83	1.04E+07	38.58
Q9Y446	PKP3	83.98	14.81	4.74E+06	87.03
Q96PK6	RBM14	79.79	22.42	9.83E+06	69.45
O15371	EIF3D	79.31	9.85	8.07E+06	63.93
A0A1W2P	HNRNPU	79.21	13.46	7.90E+06	36.40
B1ANR0	PABPC4	76.38	11.54	1.48E+07	67.93
E5RGV0	HNRNPH1	76.05	36.77	1.13E+07	17.50
A0A3B3IR	CTPS1	74.29	15.07	3.88E+06	63.68
F5GWF6	CCT2	72.44	17.17	3.92E+06	56.77
P61221	ABCE1	72.21	11.85	4.65E+06	67.27
Q8WWM7	ATXN2L	70.07	14.14	6.36E+06	113.30
O75531	BANF1	68.65	42.70	5.80E+07	10.05

Q0VGD6	HNRPR	68.38	11.04	1.24E+07	67.82
Q5T6W2	HNRNPK	64.94	16.62	9.01E+06	41.78
Q8NC56	LEMD2	64.62	11.53	4.80E+06	56.94
A0A024R7	LOC388524	61.23	17.59	1.17E+07	22.00
P25705	ATP5F1A	60.12	8.68	4.73E+06	59.71
Q15365	PCBP1	59.87	11.52	8.49E+06	37.47
Q13151	HNRNPA0	59.43	19.02	1.68E+07	30.82
P12236	SLC25A6	59.37	18.12	2.88E+07	32.85
P62805	HIST1H4A	59.27	33.01	3.19E+07	11.36
P62826	RAN	58.44	35.65	3.84E+07	24.41
Q14222	EEF1A	58.44	31.72	2.03E+08	24.18
M4VP52	APOBEC3C	57.65	26.84	1.55E+07	22.78
F5H6M0	CPSF7	55.49	10.96	5.63E+06	24.37
Q99959	PKP2	54.85	8.97	2.58E+06	97.35
Q9Y6M1	IGF2BP2	54.24	9.18	2.63E+06	66.08
F8W0P7	ATP5F1B	53.98	10.74	7.80E+06	28.37
B1AHC9	XRCC6	53.08	11.99	7.42E+06	64.24
Q06787	FMR1	52.16	15.51	1.52E+07	71.13
A0A024QZ	CDC2	52.15	34.17	4.00E+06	27.49
B2RBA6	MCM7	50.96	11.82	3.52E+06	81.23
X6R700	CHTOP	50.75	13.00	1.30E+07	23.65
P49915	GMPS	48.81	12.84	5.31E+06	76.67
Q01813	PFKP	48.01	10.33	8.12E+06	85.54
P08237	PFKM	47.04	9.62	2.83E+06	85.13
Q3MHD2	LSM12	46.66	13.85	7.87E+06	21.69
P26641	EEF1G	44.77	10.76	9.37E+06	50.09
P13010	XRCC5	44.42	14.62	1.72E+07	82.65
Q15149	PLEC	44.07	2.88	6.65E+06	531.47
P08670	VIM	43.63	12.66	3.72E+06	53.62
F8VTQ5	HNRNPA1	43.28	32.41	4.92E+06	16.53
H0YH87	ATXN2	41.08	8.14	2.46E+06	90.11
A0A286YF	PHGDH	40.81	9.70	3.66E+06	55.90
Q9NVI7	ATAD3A	40.57	15.62	8.42E+06	71.32
P51116	FXR2	39.77	16.34	1.74E+07	74.18
B0QY90	EIF3L	37.35	5.58	1.92E+06	55.13
A0A024R6	PKM2	37.29	17.97	4.62E+06	37.53
H0Y4R1	IMPDH2	37.05	14.89	3.20E+06	51.04
Q6PKI6	YBX1	36.92	7.14	1.29E+07	29.36
Q5T9A4	ATAD3B	36.73	14.81	8.42E+06	72.53
Q9BRZ2	TRIM56	36.60	8.48	8.51E+06	81.44
A0A090N8	GARS	35.73	8.76	6.43E+06	77.48
F8WJN3	CPSF6	35.04	11.30	5.90E+06	52.24
Q86V81	ALYREF	34.67	11.28	8.51E+06	26.87
Q8N884	CGAS	34.60	14.18	3.12E+06	58.78
A0A024R1	JUP	34.33	6.71	6.08E+06	81.67
D6RF44	HNRNPD	33.82	12.61	2.53E+07	12.55
A0A024R5	MARK2	33.60	7.24	1.07E+06	77.58
F8WAE5	EIF2A	32.86	9.48	2.85E+06	64.41
D3DUZ3	IFI16	31.08	11.32	1.90E+06	82.37
B4DWZ4	FEN1	30.65	7.85	5.54E+06	38.77
Q8TES4	FLJ00119	28.73	2.68	3.57E+06	153.50
Q8IY67	RAVER1	27.00	11.22	3.98E+06	63.84
Q8IW76	EIF2AK2	26.25	7.41	4.36E+06	57.58
O43776	NARS	25.83	7.48	4.34E+06	62.90
Q8NBJ5	COLGALT1	24.97	7.72	2.92E+06	71.59
F8VVM2	SLC25A3	24.60	3.70	8.61E+06	36.14
A0A087X2	SRSF3	24.08	24.21	4.52E+07	10.31
Q6PG44	HMMR	24.01	8.65	1.49E+06	71.15

P54886	ALDH18A1	23.97	4.15	2.48E+06	87.25
P10599	TXN	23.80	37.14	2.77E+06	11.73
O15187	TIAL1	23.66	16.98	1.55E+07	29.63
Q96PK6	RBM14	22.90	6.73	4.98E+06	69.45
E9PLA9	CAPRIN1	22.76	18.28	4.40E+06	20.22
Q96BS4	FBL	21.84	12.69	6.74E+06	28.43
E9PBF6	LMNB1	21.60	17.83	6.67E+06	44.62
K7EJ44	PFN1	20.36	13.46	7.95E+06	11.38
E7EPN9	PRRC2C	20.25	1.70	2.95E+06	308.59
O76094	SRP72	19.94	4.17	2.13E+06	74.56
Q9Y285	FARSA	19.83	4.92	3.69E+06	57.53
O95758	PTBP3	19.81	4.71	2.81E+07	59.65
Q9BV61	TRAP1	19.78	5.58	1.38E+07	79.31
A0A0U1R(	RBM39	19.32	16.56	2.26E+06	17.67
E9PLJ3	CFL1	18.72	39.24	1.44E+07	9.08
Q53SQ6	PTD004	18.31	10.33	2.09E+06	24.79
P13667	PDIA4	18.28	5.58	1.15E+06	72.89
Q05DV5	ZC3HAV1	18.16	6.71	1.18E+06	49.22
H3BR35	GSPT1	17.61	6.32	1.94E+06	52.91
A0A3B3IU(	COL17A1	17.39	29.51	1.18E+06	12.97
Q6ZNL4	FLJ00279	17.00	4.44	1.68E+06	65.73
Q9H9A7	RMI1	16.67	6.56	1.39E+06	70.10
O00429	DNM1L	16.62	5.71	1.24E+06	81.83
P09211	GSTP1	15.92	17.14	8.75E+06	23.34
K7EN82	NMT1	15.70	61.11	1.50E+06	8.16
Q6NT15	STON2	15.49	1.00	2.85E+07	100.88
O75131	CPNE3	14.65	5.21	3.61E+06	60.09
K7EJB9	CALR	14.23	14.57	3.07E+06	28.41
G3V4M8	HNRNPC	14.02	34.85	7.18E+06	7.28
F2Z2V6	QARS	13.98	16.00	1.51E+06	10.74
H0Y8T4	MATR3	13.64	8.38	1.28E+06	37.76
A5PLK7	RCC2	13.47	4.81	2.72E+06	49.65
A0A0C4D(	APEX1	13.41	19.18	3.94E+06	15.93
A0A0A0MF	PRDX1	13.19	21.65	5.00E+06	10.67
Q15046	KARS	13.05	6.37	2.11E+06	68.00
K7EKU4	KCNAB2	13.02	8.20	6.71E+06	12.93
P16403	HIST1H1C	12.62	5.63	1.57E+07	21.35
G3V5L5	PRMT5	12.51	11.83	2.60E+06	19.60
H3BMZ1	HACD3	12.27	10.07	1.39E+06	16.15
H0YEC5	EIF4G2	12.23	7.79	2.85E+06	27.10
A1L407	HIST1H1T	11.75	5.31	2.01E+06	22.02
P78527	PRKDC	11.68	1.02	1.17E+06	468.79
P00558	PGK1	11.55	6.24	6.82E+06	44.59
A0A1B0G\	HSD17B12	11.35	5.07	3.11E+06	30.34
Q15181	PPA1	11.31	14.53	6.77E+06	32.64
Q96AG4	LRRC59	11.05	8.14	1.26E+07	34.91
A0A0D9SE	YBX3	10.38	11.96	3.13E+06	10.53
Q15785	TOMM34	10.27	6.47	5.05E+06	34.54
I3L3C6	BAIAP2	10.02	13.43	2.05E+06	23.50
A0A024QY	EIF3C	10.00	5.86	7.14E+05	69.22

Supplemental Table 6. Translation initiation factors according to the LC-MS/MS da

Accession	GENE	Score	Area	Coverage	MW [kDa]
E7EX73	EIF4G1	142.4	1.74E+07	12.95	158.5
O15371	EIF3D	79.31	8.07E+06	9.85	63.9
B0QY90	EIF3L	37.35	1.92E+06	5.58	55.1
F8WAE5	EIF2A	32.86	2.85E+06	9.48	64.4
Q8IW76	EIF2AK2	26.25	4.36E+06	7.41	57.6
H0YEC5	EIF4G2	12.23	2.85E+06	7.79	27.1
A0A024QYX7	EIF3C	10	7.14E+05	5.86	69.2
